# A Review on Host-*Leptospira* Interactions: What We Know and Future Expectations

**DOI:** 10.3389/fcimb.2021.777709

**Published:** 2021-11-25

**Authors:** Brenda B. Daroz, Luis G. V. Fernandes, Maria F. Cavenague, Leandro T. Kochi, Felipe J. Passalia, Maria B. Takahashi, Edson G. Nascimento Filho, Aline F. Teixeira, Ana L. T. O. Nascimento

**Affiliations:** ^1^ Laboratorio de Desenvolvimento de Vacinas, Instituto Butantan, Avenida Vital Brazil, Sao Paulo, Brazil; ^2^ Programa de Pos-Graduacao Interunidades em Biotecnologia, Instituto de Ciencias Biomedicas, Universidade de São Paulo, Sao Paulo, Brazil

**Keywords:** leptospiral proteins, host-pathogen interactions, extracellular matrix components, leptospiral mutagenesis tools, cadherins, components of complement system, plasminogen-plasmin, fibrinogen,

## Abstract

Leptospirosis is a widespread zoonosis caused by pathogenic *Leptospira* spp. It is considered a neglected infectious disease of human and veterinary concern. Our group has been investigating proteins annotated as hypothetical, predicted to be located on the leptospiral surface. Because of their location, these proteins may have the ability to interact with various host components, which could allow establishment of the infection. These proteins act as adherence factors by binding to host receptor molecules, such as the extracellular matrix (ECM) components laminin and glycosaminoglycans to help bacterial colonization. *Leptospira* also interacts with the host fibrinolytic system, which has been demonstrated to be a powerful tool for invasion mechanisms. The interaction with fibrinogen and thrombin has been shown to reduce fibrin clot formation. Additionally, the degradation of coagulation cascade components by secreted proteases or by acquired surface plasmin could also play a role in reducing clot formation, hence facilitating dissemination during infection. Interaction with host complement system regulators also plays a role in helping bacteria to evade the immune system, facilitating invasion. Interaction of *Leptospira* to cell receptors, such as cadherins, can contribute to investigate molecules that participate in virulence. To achieve a better understanding of the host-pathogen interaction, leptospiral mutagenesis tools have been developed and explored. This work presents several proteins that mediate binding to components of the ECM, plasma, components of the complement system and cells, to gather research achievements that can be helpful in better understanding the mechanisms of leptospiral-host interactions and discuss genetic manipulation for *Leptospira* spp. aimed at protein function validation.

## Introduction

Leptospirosis is considered a neglected infectious disease of human and veterinary concern. The genus *Leptospira* includes both pathogenic and saprophytic species. The pathogenic group includes the causative agents of leptospirosis disease, while the saprophytic group consists of free-living non-disease-causing organisms. Leptospires can be genetically classified into 4 groups: P1 (pathogenic), P2 (intermediate) and S1 and S2 (saprophytic) (Vincent et al., 2019). They are also serologically divided, regarding serogroup and serovar status, associated with the antigenic heterogeneity of exposed lipopolysaccharides (LPSs) (Bharti et al., 2003). To date, more than 300 pathogenic serovars have been identified (Adler and de la Peña Moctezuma, 2010; Vincent et al., 2019). Human infection occurs mainly through direct contact with the urine or other biological fluids of infected animals or *via* indirect contact with contaminated soil or water (Levett, 2001; Bharti et al., 2003; Levett, 2015).

After contact with damaged skin or mucosa, pathogenic leptospires can rapidly penetrate and breach host biological barriers, being able to survive serum complement killing. They can reach target organs such as the liver, lungs and mainly the kidneys *via* the proximal tubules, within 1 hour of infection (Bharti et al., 2003), showing their high invasive potential (Haake and Levett, 2015).

The vaccines available for veterinary use are based on inactivated whole-cell or membrane preparations of pathogenic leptospires. These types of vaccines confer protective responses through, but not exclusively, the induction of antibodies against leptospiral LPS (de la Peña-Moctezuma et al., 1999; Naiman et al., 2002; Adler and de la Peña Moctezuma, 2010). However, these vaccines are not able to induce long-lasting protection and do not provide cross-protective immunity against leptospiral serovars not included in the vaccine preparation. A broad spectrum, cost-effective vaccine against leptospirosis is being pursued.

The number of genomes for which complete sequencing information is available has increased exponentially in the past two decades, including *Leptospira* spp. The available sequences combined with bioinformatics tools and DNA recombinant techniques have allowed the prediction of proteins *in silico* and their production in the laboratory, regardless of their abundance and without the need for manipulating the microorganism of study *in vitro* (Sette and Rappuoli, 2010). This has increased our understanding of the leptospiral pathogenic pathways, and the virulence factors involved, which many research groups have extensively investigated.

In the last years, several studies have revealed some outer membrane proteins of *L. interrogans* acting as adherence factors by binding to host receptor molecules. They can interact with components of the extracellular matrix (ECM) of host cells, such as laminin and glycosaminoglycans (GAGs). There are several ECM-binding proteins that potentially contribute to the leptospiral infection process (Vieira et al., 2014; Fernandes et al., 2016b). These leptospiral proteins also interact with plasma components such as plasminogen, plasmin, fibrinogen and thrombin. Another observed mechanism is the ability of these bacteria to interact with host complement system components such as C4b-binding protein (C4BP), factor H (FH), vitronectin and terminal complement components C7, C8 and C9, enabling them to survive serum attack (Cinco and Bandi, 1983; Meri et al., 2005; Verma et al., 2006; Barbosa et al., 2009; Silva et al., 2016; Siqueira et al., 2017). The process of how these interactions occur and their consequences are detailed throughout this article.

Identification and characterization of proteins that mediate the interactions with host components are essential for the understanding of leptospiral pathogenesis. Our research group has been particularly interested in proteins annotated as hypothetical, predicted to be located on *Leptospira*’s surface. Using these criteria, we gathered several leptospiral proteins that can potentially mediate the attachment of the bacteria to host components including ECM, plasma, complement system and host cells. Some of them are multifunctional, capable of binding to more than one component. The aim of this study was to put together research achievements that are helpful for further understanding the surface-exposed proteins that mediate leptospiral-host interactions and to ponder their possible significance for bacterial pathogenesis, as well discuss available genetic tools for the manipulation of *Leptospira* spp., with the aim of revealing protein function.

## Binding of *Leptospira* to ECM and Cadherins

### Laminin and E-Cadherin

Adhesive molecules present in bacterial systems can be divided into fimbriae and adhesins, the latter are capable of mediating bacterial adhesion to different elements on the surface of host cells and ECM (Pizarro-Cerdá and Cossart, 2006; Kline et al., 2009). Adhesins can be characterized as virulence factors, since they are responsible for the first steps of infection, contributing to the pathogenesis of various bacteria. Pathogenic *Leptospira* spp. have a great ability to promote infection because of their capacity to survive outside the host and the large number of susceptible mammals. One of the invasion strategies would be bacterial adhesion that recognizes components of the ECM and cell receptors such as laminin and e-cadherin, followed by cell invasion and colonization. Laminin is an adhesion glycoprotein present in the ECM of host cells, being found mainly in the basement membranes (Durbeej, 2010). Cadherins are extracellular calcium-dependent adhesion glycoproteins responsible for the formation of adherens junctions that enable the intercellular adhesion (Gallin, 1998). The structure of cadherins consists of and extracellular domain composed by five cadherin repeats responsible for Ca2^+^ binding, a transmembrane domain and a conserved intracellular domain (Marie et al., 2014).

Attachment of *L. interrogans* to laminin was demonstrated by microscopy in 2006 (Barbosa et al., 2006). Since then, several leptospiral proteins have been reported as laminin-exclusive ligands, and others have a broader range of host ligands. Lsa27 and LIC12796 are adhesins that, among all the possible components assayed, bind exclusively to laminin (Longhi et al., 2009; Lima et al., 2013). OmpL47 is an adhesin that binds to laminin, collagen III, fibronectin, aortic elastin and fibrinogen (Pinne et al., 2010). LigB is another broad-spectrum binding adhesin that interacts with collagen I, III and IV, laminin, fibronectin, elastin, tropoelastin, heparin, fibrinogen, FH, FHL-1, FHR-1 and C4bp (Choy et al., 2007; Lin et al., 2009; Castiblanco-Valencia et al., 2012; Ching et al., 2012). There are many leptospiral proteins described in the literature as laminin-binding (Choy et al., 2007; Atzingen et al., 2008; Hoke et al., 2008; Atzingen et al., 2009; Longhi et al., 2009; Oliveira et al., 2010; Vieira et al., 2010; Mendes et al., 2011; Domingos et al., 2012; Fernandes et al., 2012; Siqueira et al., 2013; Lima et al., 2013; Fernandes et al., 2014; Domingos et al., 2015; Passalia et al., 2020a).

Evangelista et al. (2014a) demonstrated that *L. interrogans* strongly binds to cadherin [vascular endothelial (VE-cadherin), epithelial (E-cadherin), neural (N-cadherin) and placental (P-cadherin) (Navarro et al., 1998; Prozialeck et al., 2004)]. There are a few *L. interrogans* proteins that have been described as cadherin-binding. For example, LIC11574 and LIC13411 are recombinant proteins that bind tightly to VE-cadherin (Evangelista et al., 2014b). The recombinant protein LIC10879, called Lsa16, interacts with E-cadherin, and when the protein is subjected to heat denaturation, binding increases. It has been suggested that unexposed amino acids on the secondary surface of Lsa16 also participate in this interaction (Pereira et al., 2017). The recombinant proteins LIC11711 and LIC12587 bind to laminin and E-cadherin, in addition to interacting with the fibrinolytic system (Kochi et al., 2019). It has been reported that virulent *L. interrogans* was able to maintain adhesion in renal proximal tubule epithelial cells, resulting in the E-cadherin cleavage and later its endocytosis with the release of the N-terminal fragment (cadherin domain repeats) into the extracellular medium (Sebastián et al., 2021).

**Figure 1:**
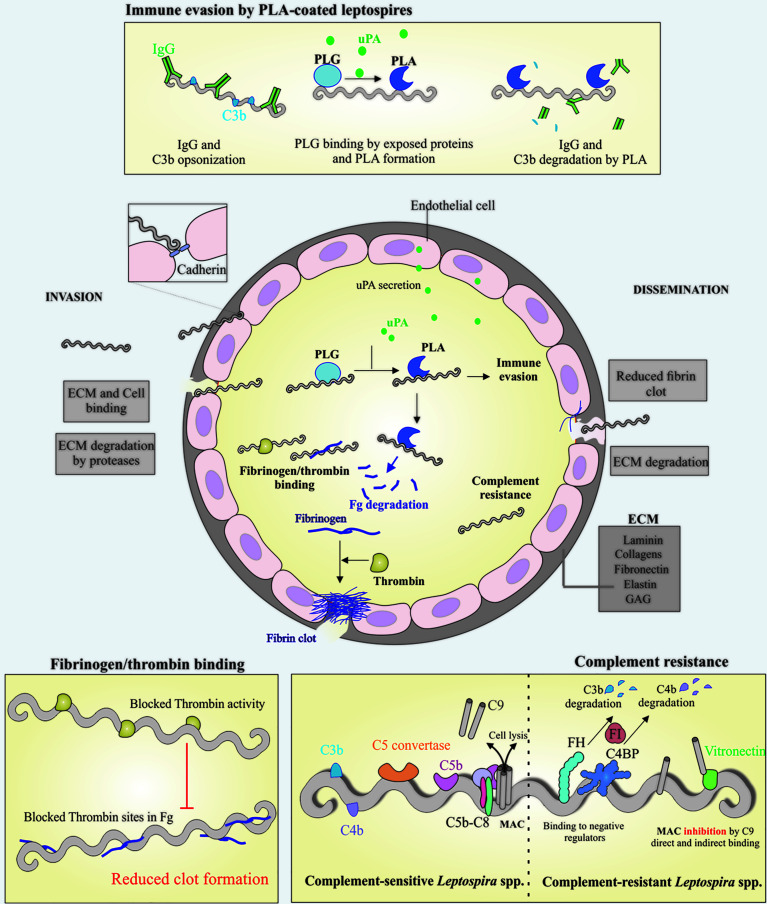
Schematic view of colonization, invasion and evasion mechanisms displayed by pathogenic *Leptospira*. Leptospires can penetrate the host *via* breached skin or intact mucosa, taking advantage of many surface exposed proteins that are able to interact with a broad range of host components, including the extracellular matrix (ECM) components and glycosaminoglycans (GAGs). During the invasion process, leptospires can directly bind to ECM components and cell receptors, as cadherins, the latter favoring cell-cell integrity disruption (center). Leptospires interact with host plasminogen (PLG) (top and center) and induce the endothelial secretion of urokinase-type PLG activator (uPA), which in turn converts leptospires-bound PLG to its active form, plasmin (PLA). The latter, a broad-spectrum serine protease, is capable of degrading ECM components and immune mediators, as IgG and C3b, reducing opsonophagocytosis (top). One of the host mechanisms to block pathogen dissemination to other sites after endothelial lesion is the formation of fibrin clot, as a result of fibrinogen (Fg) cleavage by thrombin. In addition to Fg degradation by PLA, pathogenic *Leptospira* can also bind both Fg and thrombin, causing a bilateral obstruction of the fibrin clot reaction, favoring the dissemination step (left bottom), in association with ECM degradation by endogenous proteases and surface-associated PLA (center). Once in the bloodstream, leptospires must overcome one of the first lines of host defense, the complement, and this is achieved by a multitude of mechanisms, including binding to the negative complement regulators Factor H (FH) and C4 binding protein (C4BP), which participate in the degradation of C3b and C4b, respectively. Binding to terminal components C7, C8, C9 and vitronectin, would decrease membrane attack complex (MAC) formation (right bottom). Taken together, it is anticipated that these mechanisms will facilitate invasion and dissemination of *Leptospira* through the hosts.

The leptospiral proteins that exhibit features of binding to laminin, cadherin and other host ligands are listed in **Supplementary Table 1**. It is anticipated that these proteins are possibly virulence factors for the maintenance of adhesion and infection processes of pathogenic leptospires in host cells. It is observed that *Leptospira*, like other pathogens (Isaacs, 1994; Breiner et al., 2009), has adhesin redundancy features, which is probably part of their invasion strategy. **Figure 1** depicts the interactions of *Leptospira* with host components, cells and possible consequences.

### Proteoglycans and Glycosaminoglycans in *Leptospira* Adhesion

Proteoglycans (PG) are complex macromolecules located in various animal tissues and have a broad distribution, as they are found within cells and at the cell membrane surface and ECM. They are composed of two structures: a core protein and long linear polysaccharides chain referred to as glycosaminoglycan (GAG). GAGs are composed of disaccharide repeats, usually hexoamine and uronic acid, where both units can be sulfated, increasing the PG density (Hay, 1991). Sulfation and composition of GAGs’ backbone influence their binding to several proteins and molecules with signal function, such as growth factors, cytokines, chemokines, morphogens, and enzymes (García et al., 2016). In the extracellular region, GAGs can modulate signaling by binding to those components and presenting them to their active site, acting in various cell processes such as cell adhesion, migration, proliferation, differentiation and morphogenesis, ECM assembly, tissue repair and inflammation (García et al., 2016). GAGs can also bind to microbial pathogens, an important step for bacterial adhesion to the host to facilitate invasion and colonization. Several studies have shown that mainly heparan sulfate but also chondroitin and dermatan sulfate is an important ligand for bacteria, viruses and parasites (Rostand and Esko, 1997; Shi et al., 2021).

The binding of *Leptospira* to PG and GAGs still lacks understanding about which adhesins are involved in the bacterial interaction with those components. It is already known that *Leptospira* can bind GAGs, and the binding pattern reveals that the connection is more efficient with chondroitin sulfate B (also known as dermatan sulfate) and C than heparan sulfate (Breiner et al., 2009). Contrasting with the spirochete *Borrelia burgdorferi, Leptospira* can bind to chondroitin sulfate C (Isaacs, 1994; Breiner et al., 2009). The influence of sulfation and polymer size was assessed by using dextran sulfate of different molecular weights, and it was shown that *Leptospira* had higher affinity to high molecular weight dextran sulfate. Therefore, the sulfation and size of PG polysaccharide chains are important characteristics for *Leptospira* attachment *via* GAG.

Breiner et al. (2009) and Martinez-Lopez et al. (2010) used mammalian cell cultures deficient in PG or mutant cell lines or α-galactosidase or β-xyloside to decrease cellular GAG levels; they showed that the adhesion of *L. interrogans* serovar Canicola and serovar Copenhageni to cells was partially inhibited. These results suggest that PG and GAGs play a role in *Leptospira* attachment to epithelial and endothelial cells; however, other receptors are also involved (Breiner et al., 2009; Martinez-Lopez et al., 2010). The adhesins LipL32, Loa22, OmpL1, p31/LipL45 and LenA were the first proteins described as GAG-binding proteins. From this group of proteins, only OmpL1 showed binding to heparin/heparan sulfate and chondroitin sulfate ABC (Robbins et al., 2015) (**Figure 1**). LipL21 and LipL41, lipoproteins that are among the most expressed in the outer membrane, were also found to bind to GAGs. LipL21 showed a broad binding profile, by interacting with heparin/heparan sulfate and chondroitin sulfate, while LipL41 bound effectively to chondroitin 4 sulfate (Takahashi et al., 2021).

## Cell Interactions and Adhesion

The adhesion of *Leptospira* to cell culture models has been investigated to examine localization in the host, adhesion characterization and signaling modifications and to analyze receptors and adhesins that participate in virulence. From the 1960s to 1990s, studies focused on determining the localization and cytotoxicity of *Leptospira* strains. *In vitro* cell culture started to be assessed using primary kidney cell culture, when studies showed that *L. interrogans* serovar Pomona bound more to fibroblasts than epithelial cells, and it was also observed that fibroblasts detached from the surface of flasks while epithelial cells remained adhered (Harrington and Sleight, 1966; Miller et al., 1966). Subsequently, several studies using cells from kidney proximal tubules showed bacterial adhesion to microvilli of those cells (Miller and Wilson, 1967; de Martino et al., 1969; Marshall, 1974; Sterling and Thiermann, 1981).

Localization assays not only referred to tissue specificity but indicated in which part of cell the interactions occurred. The first results suggested that *Leptospira* could be an intracellular pathogen in cell culture, as bacteria were found in the cytoplasm in microscopy assays (Rose et al., 1966; Vinh et al., 1984; Thomas and Higbie, 1990). However, an assay using translocation of polarized MDCK (Madin-Darby canine kidney) monolayer cells showed that the bacteria were invasive but not intracellular, and they were not found in intercellular junctions (Barocchi et al., 2002).

Virulent and saprophytic strains were compared regarding adherence to MDCK, L929 and other cultured kidney cells, as demonstrated by microscopy, and the virulent strains more than the saprophytic ones were found to be bound to the cells, while nonspecific adherence to plastic and glass surfaces occurred with the saprophyte *L. biflexa* (Tsuchimoto et al., 1984; Vinh et al., 1984; Ballard et al., 1986; Ito and Yanagawa, 1987). Later, during the 1990s, adhesion to epithelial and endothelial cells was quantified by radiolabeled bacteria, showing that pathogenic strains bound 1.8 to 5 times more than the saprophytic strains (Thomas and Higbie, 1990). Pathogenic *Leptospira* binding to PMN (polymorphonuclear) leukocytes and CHO (Chinese hamster ovary) mutants for Mac-1 (the CR3 integrin) was also demonstrated, indicating bacterial binding *via* integrins (Cinco et al., 2002). One study compared *L. interrogans* serovar Portlandvere and *L. borgpetersenii* serovar Jules in binding to HEp-2 (human epithelial) cells under different cell treatments (Andrade and Brown, 2012). Interestingly, Breiner et al. (2009) and Evangelista et al. (2014a) showed that *L. interrogans* bound more to cells than to ECM produced by cultured epithelial and endothelial cells (Breiner et al., 2009; Evangelista et al., 2014a).

The evaluation of cytotoxicity of *Leptospira* was assessed by the cytopathic effects induced by bacterial culture supernatant in cells (Miller et al., 1970; Yam et al., 1970; Finn and Jenkin, 1973). Other toxic effects of intact bacteria or membrane and secreted proteins were then observed in cell culture. Hemolysin SphH was able to form pores in erythrocytes, and there was lactate dehydrogenase release after 2 hours of incubation and cell lysis after 6-8 hours when using Vero, A529, H1299 and L132 cells (Lee et al., 2002). LipL32 showed the same cytotoxic profile when incubated with ECV304 cells by the release of lactate dehydrogenase and nitric oxide (Huang et al., 2008).

There are also several studies reporting an increase in PMN cell adherence and receptors in HUVEC (human umbilical vein endothelial) cells after stimulus with pathogenic bacteria, suggesting involvement in the inflammatory processes activation and host defense in vascular endothelium (Dobrina et al., 1995). Both virulent and saprophytic *Leptospira*, and the proteins LIC10365, LIC10507, LIC10508, LIC10509 and LIC12690 were also capable of stimulating HUVEC cells, as assessed by the increase in E-selectin and ICAM-1 receptors, which are involved in cell-cell and cell-ECM adherence and recruitment and migration of neutrophils to vascular endothelium (Vieira et al., 2007; Gómez et al., 2008; Atzingen et al., 2009). Another study observed an increase in von Willebrand factor when HUVEC cells were incubated with virulent bacteria, but no upregulation of E -selectin or ICAM-1 (Goeijenbier et al., 2015). The methods used were FACS and ELISA, which can produce differences in the detection of the receptors.

Modification of the cytoskeleton of cells was found in microarray and immunofluorescence using endothelial cells and virulent strains, showing a decrease in the expression of β-actin and of proteins involved in focal adhesions, leukocyte migration and ECM interaction pathways, suggesting that the virulent strain promotes actin remodeling and detachment of cells from ECM (Martinez-Lopez et al., 2010). The assays using immunofluorescence of endothelial cells were further investigated, showing morphological disruptions, as found in ZO-1 in tight junctions, and a decrease in the levels of VE-cadherin and catenins in adherence junctions was detected, indicating the VE-cadherin—catenin complex as a primary target for pathogenic *L. interrogans* (Sato and Coburn, 2017).

Receptors for adhesion to epithelial and endothelial cells were identified by assays using enzymes, lectins, integrins and saccharides (Cinco et al., 2002; Andrade and Brown, 2012). Assays using protein array technologies were an interesting tool to identify and screen receptors important to pathogen adherence. The evaluation of receptors by mass spectroscopy and protein array identified the family of cadherins as receptors for *Leptospira*, and in this work, several cell lines were evaluated and showed binding (Evangelista et al., 2014a). In phage display assays, LIC11574 showed binding to epithelial and endothelial cells, and also bound to VE-cadherin (Evangelista et al., 2014b), and LIC12976, a laminin-binding protein, bound to fibroblasts and epithelial cells (Lima et al., 2013). LIC10831 was also assessed as an E- and VE-cadherin ligand using different cell lines, including CHO mutants expressing the receptor and endothelial cells (Eshghi et al., 2019). The terminal repeats of the proteins LigA and LigB, which interacted with the gelatin binding domain of fibronectin, were able to bind to MDCK cells and inhibited the ligation of L. interrogans serovar Pomona to the monolayers (Lin et al., 2010). Also, LigB and a mutant of *L. biflexa* expressing LigA showed binding to human embryonic lung cells and these interactions were blocked in the presence of human tropoelastin up to 68% and 61%, respectively (Hsieh et al., 2017).

The major proteins from *Leptospira*, previously characterized as adhesins (LipL32, Loa22, OmpL1, p31/LipL45 and LenA) were evaluated in epithelial and endothelial cells, and only OmpL1 displayed a significant difference in binding to Hep-2 and EA.hy926 cells (Robbins et al., 2015).

## Binding of *Leptospira* to Plasma Components

### Leptospiral Proteins That Bind to Plasminogen

The interaction of *Leptospira* with the host fibrinolytic system has been demonstrated to be a powerful tool for invasion mechanisms. Plasmin is the major component of the fibrinolytic system, a broad-spectrum serine protease that is activated after plasminogen cleavage by tissue-type (tPA) or urokinase-type (uPA) plasminogen activator. Plasminogen is found in human tissues and plasma in high amounts; its structure contains five kringle domains, which mediate binding to several ligands *via* their lysine residues (Castellino and Ploplis, 2005). The ability of *Leptospira* spp. to bind plasminogen on its surface and convert it to plasmin in the presence of an exogenous activator, can provide leptospires with certain advantages. *Leptospira* associated with plasmin have the capacity to cleave ECM proteins and degrade complement components, such as C3b and IgG, interfering with the deposition of these molecules on the bacterial surface and consequently disrupting the opsonophagocytosis process, which facilitates bacterial immune evasion (**Figure 1**) (Vieira et al., 2009; Vieira et al., 2011; Vieira et al., 2013; Verma et al., 2020)

In the last few years, several proteins experimentally described as located on the *Leptospira* surface have been identified as plasminogen-binding. Interactions have been demonstrated to occur mainly *via* the lysine residues in proteins and plasminogen kringle domains, since the interactions were inhibited by a lysine analog, as observed by *in vitro* assay (Domingos et al., 2012; Teixeira et al., 2015; Fernandes et al., 2016b; Vieira and Nascimento, 2016; Pereira et al., 2017; Passalia et al., 2020a; Passalia et al., 2021). Among many proteins already identified as a plasminogen receptor, the major outer membrane lipoproteins LipL32, LipL21 and LipL41 and the transmembrane protein OmpL1 are included (Fernandes et al., 2012; Vieira et al., 2010; Takahashi et al., 2021). As reported for the bacteria, plasminogen bound to recombinant proteins is converted to active plasmin in the presence of an exogenous activator. Also, proteins such as rLIC11711, rLIC13259, Lsa24.9, rLIC13086 and LipL41 were able to acquire plasminogen from human serum, suggesting the viability of these interactions under physiological conditions and their possible role in leptospiral virulence (Cavenague et al., 2019; Kochi et al., 2019; Rossini et al., 2020; Passalia et al., 2021; Takahashi et al., 2021). Leptospiral immunoglobulin-like proteins, known as Lig proteins, have also been identified as plasminogen-binding. It was observed that plasminogen bound to these proteins was converted to active plasmin and able to degrade fibrinogen and complement proteins C3b and C5 (Oliveira et al., 2013; Castiblanco-Valencia et al., 2016)

Although the major proteins identified as plasminogen-binding are described as being outer membrane or secreted proteins, cytoplasmic proteins have also been identified as plasminogen-binding (Vieira et al., 2012; Nogueira et al., 2013). Enolase is described as a metabolic enzyme, but in *Leptospira*, it was shown to be secreted and have the ability to interact with plasminogen (Nogueira et al., 2013). The role of cytoplasmic proteins in host-pathogen interactions is still undefined, but proteins such as DnaK, glutamine synthetase and acetyltransferase were also identified as plasminogen ligands (Vieira et al., 2012). It is speculated that, at some point, these proteins are exported to the bacterial surface or after cell lysis these proteins could find plasminogen, helping surviving cells to disseminate in host tissues.

Most of the plasminogen-binding proteins identified until now do not display exclusive interaction with this component. They have the ability to interact with other host components, which characterize them as multifunctional molecules. In contrast, some proteins such as LipL46, Lp30 and Lp49 show plasminogen-exclusive binding properties (**Figure 1**) (Vieira et al., 2010; Oliveira et al., 2011; Santos et al., 2018). The reason why some proteins bind exclusively to plasminogen and others do not is still unclear. Thus, it is possible that the multiple binding characteristics observed by surface membrane proteins may contribute to leptospiral pathogenesis. The main features of proteins identified as plasminogen-binding and their interactions with other host molecules are summarized in **Supplementary Table 1**.

### Leptospiral Protein Interactions With Fibrinogen and Thrombin

Fibrinogen is a homodimeric glycoprotein complex synthetized primarily in hepatocytes, and it circulates in plasma at high concentrations (2-5 mg/mL) in healthy individuals. In coagulation, fibrinogen is enzymatically converted to insoluble fibrin by proteolytic cleavage of N-terminal fibrinopeptides mediated by thrombin. Clot formation, stability and structure are influenced by several factors such as concentrations of anticoagulants, procoagulants, metal ions and fibrinogen-binding proteins during fibrin formation (Doolittle, 1984; Weisel, 2005; Wolberg and Campbell, 2008).

Several bacterial pathogens have mechanisms to overcome clotting in the fibrinolytic system; this can be achieved through degradation of host components by secreting proteases or using host plasminogen (Lähteenmäki et al., 2001). Fibrinogen acts as the major component in clot formation during vascular injury and tissue damage, besides stopping bacterial dissemination (Chierakul et al., 2008; Wagenaar et al., 2010). It has been reported that pathogenic *Leptospira* spp. are able to bind either fibrinogen or thrombin, promoting a bilateral obstruction, thereby reducing fibrin clot formation. Additionally, the degradation of coagulation cascade components by secreted proteases or by acquired surface plasmin could also play a role on reducing clot formation, thereby facilitating dissemination during the establishment of infection. In leptospirosis patients, activated coagulation is observed, with increased levels of fibrin degradation products and plasma fibrinogen and reduced levels of antithrombin, associated with tissue damage and vascular injury (**Figure 1**) (Oliveira et al., 2013; Fernandes et al., 2015; Fernandes et al., 2016a).

The interaction of *Leptospira* spp. with fibrinogen is mediated by several outer membrane proteins. To date, the fibrinogen-binding proteins identified include: LigA and LigB (Choy et al., 2011; Lin et al., 2011), OmpL37 (Pinne et al., 2010), Lsa33, Lsa25, Lsa30 and OmpL1 (Oliveira et al., 2013), Lsa23, Lsa36 (Siqueira et al., 2013), Lsa37 (Silva et al., 2016), rLIC10508 (Siqueira et al., 2015), Lsa25.6 and Lsa16 (Pereira et al., 2017), ErpY (Ghosh et al., 2019), rLIC10774 (Passalia et al., 2020a), and rLIC13086 (Passalia et al., 2021). The interactions with most of these proteins were found to be dose-dependent and specific. The inhibitory effect of fibrin clot formation was, however, only observed with LigB fragment 9-11 (Choy et al., 2011) and LigBCen2 (amino acids 1014–1165 of LigB) (Lin et al., 2011), Lsa33, rLIC12238, Lsa36, OmpL1, Lsa37, Lsa25.6, ErpY and rLIC13086 (**Figure 1**), and it was incomplete, reaching a maximum of 90%. Although these results differ from other bacterial fibrinogen-binding proteins, ClfA of *Staphylococcus aureus* (Liu et al., 2005) and SdrG of *Staphylococcus epidermidis* (Davis et al., 2001), leptospires may use their redundant multifunctional proteins to overcome the clotting barrier.

Leptospires can interact with different components of the fibrinolytic system during the dissemination process. The binding to thrombin, observed to a higher degree in virulent strains, followed by culture-attenuated ones, occurs *via* the substrate-binding exosite I, and it was demonstrated that fibrin clotting is inhibited (Fernandes et al., 2015). The only reported protein to bind thrombin was LIC10774, but this interaction did not block clot formation. Additionally, leptospiral BatA and the serine protease BatB proteins were able to cause a disorder in platelet aggregation, another mechanism that leptospires can overcome in blood to disseminate (Fang et al., 2018; Passalia et al., 2020b).

## Components of Complement System

The complement system is considered one of the first lines of defense against invading microorganisms because of its opsonic, inflammatory and lytic capacities. Complement effector functions result from the activation of three different pathways: classical, alternative, and/or lectin pathways (CP, AP and LP, respectively). Once activated, C5b initiates the terminal pathway and allows the association of C6 and C7 molecules. Component C7 is inserted into the lipid bilayer of the microorganism membrane and the interaction of C8 leads to stability of the C5b-7 complex. The association of several C9 molecules forms MAC, generating the C5b-9 complex and subsequently causing cell lysis (Kim and Song, 2006; Ricklin et al., 2010).

It has been shown that *L. biflexa* is rapidly killed in the presence of normal human serum (NHS), while pathogenic species are able to resist serum attack (Cinco and Bandi, 1983; Meri et al., 2005). This is due to the ability of these bacteria to interact with host complement system regulators, such as FH (Meri et al., 2005; Verma et al., 2006), C4BP (Barbosa et al., 2009), vitronectin (da Silva et al., 2015) and terminal complement components C7, C8 and C9 (**Figure 1**) (Siqueira et al., 2017).

Several leptospiral proteins have been identified as FH and C4BP receptors. Endostatin-like (Len) proteins A (LenA) and B (LenB) (Stevenson et al., 2007), EF-Tu protein (Wolff et al., 2013) and Erp-Y-like lipoprotein (Ghosh et al., 2019) were identified as ligands of FH. Among these proteins, only LenA and EF-Tu showed the ability to inactivate C3b. The interaction with C4BP was demonstrated by Lsa30 (Souza et al., 2012), rLIC10774 (Passalia et al., 2020a) and rLIC13086 (Passalia et al., 2021), but only Lsa30 was assayed and shown to mediate C4b inactivation (Souza et al., 2012). Several proteins were able to interact with both regulators and inactivate C3b and C4b, such as the LigA and LigB (Castiblanco-Valencia et al., 2012), LcpA (da Silva et al., 2015), enolase (Salazar et al., 2017) and Lsa23 (Siqueira et al., 2016; Siqueira et al., 2017). The fine mapping of the interaction between C4BP and outer membrane proteins, LigA and LigB was assessed by Breda et al. (2015). The fragments LigA7-8, LigA9-10, LigA10-11, LigB7-8, LigB9-10 and LigB11-12 were able to interact with host protein.

In addition to binding to FH and C4BP, LcpA also interfered with the complement cascade by interacting with vitronectin and preventing C9 polymerization and MAC formation (da Silva et al., 2015). In the same way, Lsa23 was also able to interact with terminal complement components C8 and C9 (Siqueira et al., 2017), while rLIC10774 (Passalia et al., 2020a) and rLIC13086 were able to bind to C7, C8 and C9, and rLIC13086 also could recruit these components directly from NHS (Passalia et al., 2021). Furthermore, rLIC11711 exhibited binding to vitronectin and C8 (Kochi et al., 2019), while rLIC12587 and rLIC13259 showed binding to vitronectin, C7, C8 and C9 (Kochi et al., 2019; Cavenague et al., 2019). The recombinant proteins were able to capture the complement system components from NHS, and inhibit MAC formation, thus possibly contributing to leptospiral immune evasion (**Figure 1**) (Cavenague et al., 2019; Kochi et al., 2019).

## Mutagenesis in *Leptospira* spp. for Protein Function Validation

**Figure 2:**
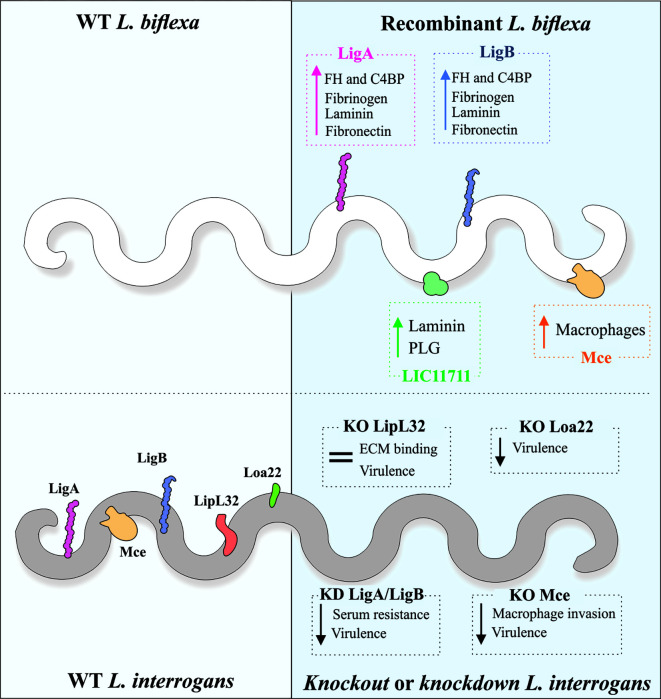
Genetic tools and mutant evaluation of *Leptospira.* As the saprophytic *L. biflexa* lacks most of the virulence-associated proteins, it is used as a surrogate for the expression of pathogen-specific proteins and gain-of-function phenotype evaluation. Increased binding to ECM and plasma components has been observed in recombinant *L. biflexa* expressing *L. interrogans* proteins. Contrarily to this strategy, knockout (KO) or knockdown (KD) in the pathogenic *L. interrogans* has been used to evaluate loss-of-function phenotypes, in comparison to the wild-type strain. Reduced virulence in animal model was observed for KD double LigA/LigB, KO Loa22 and KO Mce mutants. KO of LipL32, the major lipoprotein of pathogenic leptospires, did not alter virulence or ECM binding.

Properties displayed *in vitro* by purified recombinant protein do not necessarily reflect the native counterpart role in *Leptospira* spp. Accordingly, functional genomic and host-pathogen interaction analysis require genetic mutations in particular genes to assess the resulting phenotype (Shapiro et al., 2018). Gene *knockout* or *knockdown* in pathogenic species of *Leptospira* should ideally lead to a loss of function phenotype, which can be measured by interaction assays with purified host ligands and/or cultured cells, translocation assays or even challenge with host serum, for evaluating the outcome of leptospiral binding to complement regulators. On the other hand, the expression of pathogen-specific genes in the saprophyte *L. biflexa* has offered an alternative for studying protein function by gain-of-function phenotypes and, in some cases, has offered a complementation to results obtained by *L. interrogans* mutants (**Figure 2**).

The application of mariner-based transposon mutagenesis revealed Loa22 as a virulence factor of *Leptospira* since the transposon disruption in the *loa22* gene resulted in an avirulent mutant (Ristow et al., 2007). Likewise, disruption of the *flaA1* and *flaA2* genes or just *flaA2* resulted in reduced bacterial motility and less virulent mutant strains, indicating that motility is associated with leptospiral invasion (Lambert et al., 2012).

Mutation in the surface-exposed LruA resulted in attenuation of the virulence of *L. interrogans* compared to the wild-type strain in a hamster model of infection (Zhang et al., 2013). Interestingly, mutations in the genes encoding the *L. interrogans* serovar Manilae proteins LipL32 (Murray et al., 2009) and LipL41 (King et al., 2013), two of the most abundant and highly conserved outer membrane proteins in pathogenic *Leptospira* species, did not alter leptospiral virulence or symptoms of acute leptospirosis in infected hamsters. Accordingly, the *lipL32* mutant displayed no difference in binding to a commercially available ECM preparation, laminin or collagen type I, in comparison to a control intergenic mutant (Murray et al., 2009), highlighting the functional redundancy displayed by leptospires. Binding assays were not performed for the *lipL41* mutant (King et al., 2013).

Croda et al. (2008) performed site-directed mutation in pathogenic *Leptospira* by allelic exchange, utilizing a suicide plasmid to deliver a spectinomycin resistance cassette flanked by two “homology arms” corresponding to the *lig*B coding region. *In vitro* adherence of the *lig*B mutant to MDCK monolayers showed no difference between this and the wild-type strain. Accordingly, disruption of *lig*B did not affect virulence and persistence in animal models, probably because of functional redundancy to the *lig*A gene product.

Site-directed inactivation of the *mce* (mammalian cell entry, LA2055, homologous to LIC11859) in *L. interrogans* serovar Lai by Zhang et al., 2012 resulted in significantly diminished adherence invasion of murine J774A.1 macrophages in comparison to wild type strain; attenuation of virulence was also observed for the *mce knockout* mutant.

Pappas and Picardeau (2015) used a transposon-delivered cassette containing the *Xanthomonas* transcription activator-like effector (TALE) targeting both *lig*A and *lig*B genes, aiming the blockage of gene transcription (knockdown), thereby reducing but not abolishing the levels of LigA and LigB proteins. Though the authors did not perform any functional characterization of the mutants regarding interaction with host components, attenuation in the hamster model could be observed, indicating that both proteins are required for virulence (Pappas and Picardeau, 2015).

Concomitant and complete silencing of both LigA and LigB proteins by CRISPR-interference (CRISPRi) resulted in a drastic reduction of *L. interrogans* survival upon serum challenge, corroborating their interaction with complement regulators (Fernandes et al., 2021). In addition, this augmented serum susceptibility resulted in avirulent leptospires (Fernandes et al., 2021, manuscript in preparation), as previously demonstrated (Pappas and Picardeau, 2015).

Results obtained with mutants in *L. interrogans* agreed with the phenotypes observed by expression in the surrogate *L. biflexa*, favoring the elucidation of the complement resistance displayed by pathogenic leptospires and how LigA and LigB proteins fit in the scenario. *L. biflexa* individually expressing LigA or LigB gained the ability to sequester the negative complement regulators FH and C4BP, which retained the cofactor activity on the leptospiral surface. As a result, the recombinant *L. biflexa* displayed enhanced survival upon human serum challenge (Castiblanco-Valencia et al., 2016).

Zhang et al. (2012) used *L. biflexa* expressing the Mce protein to confirm the results obtained with the allelic exchange mutant in *L. interrogans*, showing that the recombinant bacteria displayed increased capacity for binding to murine macrophages. In addition, the heterologous expression of *lmb216* (under *lipL32* promoter) and *ligB* (under borrelial *flaB* promoter) in *L. biflexa* resulted in enhanced adhesion to fibronectin and phagocytic uptake, confirming the results obtained with the respective transposon mutants in *L. interrogans* (Toma et al., 2014).

The *L. biflexa* surrogate system was also employed to validate Lig protein binding to ECM molecules and host cells (Figueira et al., 2011). Constitutive expression of LigA driven by the borrelial *flgB* promoter resulted in enhanced adherence of the recombinant bacteria to MDCK cells, in comparison to the wild-type strain; neither LigA nor LigB expression influenced the bacterial translocation across MDCK monolayers. Recombinant *L. biflexa* expressing LigA or LigB displayed increased interaction with plasma and cellular fibronectin and laminin but not with elastin or collagens (Figueira et al., 2011), and also enhanced binding to human fibrinogen (Choy et al., 2011).

Overexpression of the pathogen-specific LIC11711 gene by genetic fusion of the coding sequence to the strong and constitutive *lipL32* promoter strengthened the adhesin properties displayed by the recombinant counterpart according to *in vitro* assays, since this protein was suggested to be involved in leptospiral binding to laminin and plasminogen (Kochi et al., 2019). *L. biflexa* expressing LIC11711 on its surface showed increased binding to laminin and plasminogen compared to the wild-type or empty plasmid-containing strains. LIC11711-bound plasminogen was capable of being converted to plasmin in the presence of uPA (Kochi et al., 2020), where this was the first time that a mutant was used to validate a leptospiral plasminogen receptor (**Figure 2**).

## Concluding Remarks

We offer here an overview of many proteins possibly involved in the pathogenesis of *Leptospira*. The interaction of these proteins with ECM components can mediate the attachment of *Leptospira* to mammalian host cells, starting the process of invasion/colonization. Some proteins bind plasminogen at the bacterial surface, which is then converted to plasmin; surface plasmin gives the bacteria proteolytic capability, contributing to the invasion process. In addition, surface plasmin prevents C3b and IgG deposition on the leptospiral surface, reducing opsonophagocytosis. Pathogenic *Leptospira* spp. can also bind fibrinogen and thrombin, causing a bilateral obstruction and reduction of fibrin clot formation, leading to possible hemorrhage foci. In addition, these bacteria can resist serum attack, which has been linked to their ability to interact with host complement system components, namely C4BP, FH, vitronectin, C7, C8 and C9, contributing to immune evasion. The adhesion of *Leptospira* to cell culture models to investigate localization in the host has contributed to determining receptors and adhesins that are involved in virulence.

We highlight the progress in the arsenal of genetic tools now available for gene knockout or knockdown in *Leptospira* spp., both pathogenic and saprophytic. These advances in confluence with the numerous data on recombinant proteins will greatly expand our understanding of the host-pathogen interaction. With the constantly increasing available data on leptospiral host-pathogen interaction, it became yet more evident how multifunctional these pathogens are, illustrated by not only the vast range of pathophysiologic mechanisms that they participate, but also by the numerous and redundant surface bacterial receptors. As future expectations, application of genetic tools to demonstrate “true” virulence determinants amongst all described leptospiral adhesins will narrow down the array of vaccine candidates. Due to the established leptospiral functional plasticity, it is anticipated that the best strategy will be merging these adhesins, proved to be required for virulence, as chimeric constructions, ultimately leading to a more rational vaccine development for controlling leptospirosis.

## Author Contributions 

All authors listed have made a substantial, direct, and intellectual contribution to the work, and approved it for publication.

## Funding

The following Brazilian agencies: FAPESP (grant 2019/17488-2), CNPq (grant 301229/2017-1) and Fundação Butantan, financially supported this work; AT, MC, LK, EF, LF, FP, MT, and BD have FAPESP fellowship (2016/11541-0; 2018/08131-0; 2018/09652-4; 2018/06201-1; 2017/06731-8; 2017/01102-2; 2017/26223-7; 2018/21959-8, respectively). The funders had no role in study design, analysis, decision to publish, or preparation of the manuscript.

## Conflict of Interest

The authors declare that the research was conducted in the absence of any commercial or financial relationships that could be construed as a potential conflict of interest.

## Publisher’s Note

All claims expressed in this article are solely those of the authors and do not necessarily represent those of their affiliated organizations, or those of the publisher, the editors and the reviewers. Any product that may be evaluated in this article, or claim that may be made by its manufacturer, is not guaranteed or endorsed by the publisher.

## References

[B1] Adler B. de la Peña Moctezuma A. (2010). Leptospira and Leptospirosis. Vet. Microbiol. 140(3–4), 287–296. doi: 10.1016/j.vetmic.2009.03.012 19345023

[B2] Andrade G. I. Brown P. D. (2012). A Comparative Analysis of the Attachment of *Leptospira Interrogans* and *L. Borgpetersenii* to Mammalian Cells. FEMS Immunol. Med. Microbiol. 65, 105–115. doi: 10.1111/j.1574-695X.2012.00953.x 22409511

[B3] Atzingen M. V. Barbosa A. S. De Brito T. Vasconcellos S. A. de Morais Z. M. Lima D. M.. (2008). Lsa21, a Novel Leptospiral Protein Binding Adhesive Matrix Molecules and Present During Human Infection. BMC Microbiol. 8 (8), 70. doi: 10.1186/1471-2180-8-70 18445272 PMC2386478

[B4] Atzingen M. V. Gómez R. M. Schattner M. Pretre G. Gonçales A. P. de Morais Z. M.. (2009). Lp95, a Novel Leptospiral Protein That Binds Extracellular Matrix Components and Activates E-Selectin on Endothelial Cells. J. Infect. 59, 264–276. doi: 10.1016/j.jinf.2009.07.010 19665803

[B5] Ballard S. A. Williamson M. Adler B. Vinh T. Faine S. (1986). Interactions of Virulent and Avirulent Leptospires With Primary Cultures of Renal Epithelial Cells. J. Med. Microbiol. 21, 59–67. doi: 10.1099/00222615-21-1-59 3512834

[B6] Barbosa A. S. Abreu P. A. E. Neves F. O. Atzingen M. V. Watanabe M. M. Vieira M. L.. (2006). A Newly Identified Leptospiral Adhesin Mediates Attachment to Laminin. Infect. Immun. 74, 6356–6364. doi: 10.1128/IAI.00460-06 16954400 PMC1695492

[B7] Barbosa A. S. Abreu P. A. E. Vasconcellos S. A. Morais Z. M. Gonçales A. P. Silva A. S.. (2009). Immune Evasion of Leptospira Species by Acquisition of Human Complement Regulator C4BP. Infect. Immun. 77, 1137–1143. doi: 10.1128/IAI.01310-08 19114549 PMC2643629

[B8] Barocchi M. A. Ko A. I. Reis M. G. McDonald K. L. Riley L. W. (2002). Rapid Translocation of Polarized MDCK Cell Monolayers by *Leptospira Interrogans*, an Invasive But Nonintracellular Pathogen. Infect. Immun. 70, 6926–6932. doi: 10.1128/IAI.70.12.6926-6932.2002 12438371 PMC132952

[B9] Bharti A. R. Nally J. E. Ricaldi J. N. Matthias M. A. Diaz M. M. Lovett M. A.. (2003). Leptospirosis: A Zoonotic Disease of Global Importance. Lancet Infect. Dis. 3, 757–771. doi: 10.1016/S1473-3099(03)00830-2 14652202

[B10] Breda L. C. D. Hsieh C.-L. Castiblanco Valencia M. M. da Silva L. B. Barbosa A. S. Blom A. M.. (2015). Fine Mapping of the Interaction Between C4b-Binding Protein and Outer Membrane Proteins LigA and LigB of Pathogenic Leptospira Interrogans. PloS Negl. Trop. Dis. 9, e0004192. doi: 10.1371/journal.pntd.0004192 26517116 PMC4627802

[B11] Breiner D. D. Fahey M. Salvador R. Novakova J. Coburn J. (2009). *Leptospira Interrogans* Binds to Human Cell Surface Receptors Including Proteoglycans. Infect. Immun. 77, 5528–5536. doi: 10.1128/IAI.00546-09 19805539 PMC2786458

[B12] Castellino F. Ploplis V. (2005). Structure and Function of the Plasminogen/Plasmin System. Thromb. Haemost. 93, 647–654. doi: 10.1160/TH04-12-0842 15841308

[B13] Castiblanco-Valencia M. M. Fraga T. R. Breda L. C. D. Vasconcellos S. A. Figueira C. P. Picardeau M.. (2016). Acquisition of Negative Complement Regulators by the Saprophyte Leptospira Biflexa Expressing LigA or LigB Confers Enhanced Survival in Human Serum. Immunol. Lett. 173, 61–68. doi: 10.1016/j.imlet.2016.03.005 26976804 PMC5437552

[B14] Castiblanco-Valencia M. M. Fraga T. R. Silva L. B. Monaris D. Abreu P. A. E. Strobel S.. (2012). Leptospiral Immunoglobulin-Like Proteins Interact With Human Complement Regulators Factor H, FHL-1, FHR-1, and C4BP. J. Infect. Dis. 205, 995–1004. doi: 10.1093/infdis/jir875 22291192

[B15] Cavenague M. F. Teixeira A. F. Filho A. S. Souza G. O. Vasconcellos S. A. Heinemann M. B.. (2019). Characterization of a Novel Protein of Leptospira Interrogans Exhibiting Plasminogen, Vitronectin and Complement Binding Properties. Int. J. Med. Microbiol. 309, 116–129. doi: 10.1016/j.ijmm.2018.12.005 30638770

[B16] Chierakul W. Tientadakul P. Suputtamongkol Y. Wuthiekanun V. Phimda K. Limpaiboon R.. (2008). Activation of the Coagulation Cascade in Patients With Leptospirosis. Clin. Infect. Dis. 46, 254–260. doi: 10.1086/524664 18171258

[B17] Ching A. T. C. Fávaro R. D. Lima S. S. Chaves A. de A. M. de Lima M. A.. (2012). *Lepstospira Interrogans* Shotgun Phage Display Identified LigB as a Heparin-Binding Protein. Biochem. Biophys. Res. Commun. 427, 774–779. doi: 10.1016/j.bbrc.2012.09.137 23044419

[B18] Choy H. A. Kelley M. M. Chen T. L. Møller A. K. Matsunaga J. Haake D. A. (2007). Physiological Osmotic Induction of *Leptospira Interrogans* Adhesion: LigA and LigB Bind Extracellular Matrix Proteins and Fibrinogen. Infect. Immun. 75, 2441–2450. doi: 10.1128/IAI.01635-06 17296754 PMC1865782

[B19] Choy H. A. Kelley M. M. Croda J. Matsunaga J. Babbitt J. T. Ko A. I.. (2011). The Multifunctional LigB Adhesin Binds Homeostatic Proteins With Potential Roles in Cutaneous Infection by Pathogenic Leptospira Interrogans. PloS One 6, e16879. doi: 10.1371/journal.pone.0016879 21347378 PMC3036719

[B20] Cinco M. Banfi E. (1983). Activation of Complement by Leptospires and its Bactericidal Activity. Zentralbl. Bakteriol. Mikrobiol. Hyg. A. 254, 261–265. 6675348

[B21] Cinco M. Cini B. Perticarari S. Presani G. (2002). Leptospira Interrogans Binds to the CR3 Receptor on Mammalian Cells. Microb. Pathog. 33, 299–305. doi: 10.1006/mpat.2002.0537 12495676

[B22] Croda J. Figueira C. P. Wunder E. A. Santos C. S. Reis M. G. Ko A. I.. (2008). Targeted Mutagenesis in Pathogenic Leptospira Species: Disruption of the LigB Gene Does Not Affect Virulence in Animal Models of Leptospirosis. Infect. Immun. 76, 5826–5833. doi: 10.1128/IAI.00989-08 18809657 PMC2583567

[B23] da Silva L. B. Miragaia L. dos S. Breda L. C. D. Abe C. M. Schmidt M. C. B.. (2015). Pathogenic Leptospira Species Acquire Factor H and Vitronectin *via* the Surface Protein LcpA. Infect. Immun. 83, 888–897. doi: 10.1128/IAI.02844-14 25534939 PMC4333444

[B24] Davis S. L. Gurusiddappa S. McCrea K. W. Perkins S. Höök M. (2001). SdrG, a Fibrinogen-Binding Bacterial Adhesin of the Microbial Surface Components Recognizing Adhesive Matrix Molecules Subfamily From Staphylococcus Epidermidis, Targets the Thrombin Cleavage Site in the Bβ Chain. J. Biol. Chem. 276, 27799–27805. doi: 10.1074/jbc.M103873200 11371571

[B25] de la Peña-Moctezuma A. Bulach D. M. Kalambaheti T. Adler B. (1999). Comparative Analysis of the LPS Biosynthetic Loci of the Genetic Subtypes of Serovar Hardjo: Leptospira Interrogans Subtype Hardjoprajitno and Leptospira Borgpetersenii Subtype Hardjobovis. FEMS Microbiol. Lett. 177, 319–326. doi: 10.1111/j.1574-6968.1999.tb13749.x 10474199

[B26] de Martino C. Bruni C. B. Bellocci M. Natali P. G. (1969). Spontaneous Leptospiral Infection of the Rat Kidney. An Ultrastructural Study. Exp. Mol. Pathol. 10, 27–38. doi: 10.1016/0014-4800(69)90046-X 5764538

[B27] Dobrina A. Nardon E. Vecile E. Cinco M. Patriarca P. (1995). Leptospira Icterohemorrhagiae and Leptospire Peptidolgycans Induce Endothelial Cell Adhesiveness for Polymorphonuclear Leukocytes. Infect. Immun. 63, 2995–2999. doi: 10.1128/iai.63.8.2995-2999.1995 7542637 PMC173407

[B28] Domingos R. F. Romero E. C. Fernandes L. G. Vasconcellos S. A. de Morais Z. M. Nascimento A. L. T. O. (2015). Novel *Leptospira Interrogans* Protein Lsa32 Is Expressed During Infection and Binds Laminin and Plasminogen. Microbiology 161, 851–864. doi: 10.1099/mic.0.000041 25627443

[B29] Domingos R. F. Vieira M. L. Romero E. C. Gonçales A. de Morais Z. M. Vasconcellos S. A.. (2012). Features of Two Proteins of *Leptospira Interrogans* With Potential Role in Host-Pathogen Interactions. BMC Microbiol. 12, 50. doi: 10.1186/1471-2180-12-50 PMC3444417 22463075

[B30] Doolittle R. F. (1984). Fibrinogen and Fibrin. Annu. Rev. Biochem. 53, 195–229. doi: 10.1146/annurev.bi.53.070184.001211 6383194

[B31] Durbeej M. (2010). Laminins. Cell Tissue Res. 339, 259–268. doi: 10.1007/s00441-009-0838-2 19693542

[B32] Eshghi A. Gaultney R. A. England P. Brûlé S. Miras I. Sato H.. (2019). An Extracellular Leptospira Interrogans Leucine-Rich Repeat Protein Binds Human E- and VE-Cadherins. Cell. Microbiol. 21, e12949. doi: 10.1111/cmi.12949 30171791 PMC7560960

[B33] Evangelista K. Franco R. Schwab A. Coburn J. (2014a). Leptospira interrogans Binds to Cadherins. PLoS Negl. Trop. Dis. 8, e2672. doi: 10.1371/journal.pntd.0002672 24498454 PMC3907533

[B34] Evangelista K. V. Hahn B. Wunder E. A. Ko A. I. Haake D. A. Coburn J. (2014b). Identification of Cell-Binding Adhesins of Leptospira interrogans. PLoS Negl. Trop. Dis. 8, e3215. doi: 10.1371/journal.pntd.0003215 25275630 PMC4183468

[B35] Fang J.-Q. Imran M. Hu W.-L. Ojcius D. M. Li Y. Ge Y.-M.. (2018). vWA Proteins of Leptospira Interrogans Induce Hemorrhage in Leptospirosis by Competitive Inhibition of vWF/GPIb-Mediated Platelet Aggregation. EBioMedicine 37, 428–441. doi: 10.1016/j.ebiom.2018.10.033 30337247 PMC6284457

[B36] Fernandes L. G. de Morais Z. M. Vasconcellos S. A. Nascimento A. L. T. O. (2015). *Leptospira Interrogans* Reduces Fibrin Clot Formation by Modulating Human Thrombin Activity *via* Exosite I. Pathog. Dis. 73, ftv001. doi: 10.1093/femspd/ftv001 25834144

[B37] Fernandes L. G. V. Hornsby R. L. Nascimento A. L. T. O. Nally J. E. (2021). Genetic Manipulation of Pathogenic *Leptospira*: CRISPR Interference (CRISPRi)-Mediated Gene Silencing and Rapid Mutant Recovery at 37°C. Sci. Rep. 11, 1768. doi: 10.1038/s41598-021-81400-7 33469138 PMC7815788

[B38] Fernandes L. G. Filho A. F. Souza G. O. Vasconcellos S. A. Romero E. C. Nascimento A. L.. (2016a). Decrease in Antithrombin III and Prothrombin Serum Levels Contribute to Coagulation Disorders During Leptospirosis. Microbiology 162 (8), 1407–1421. doi: 10.1016/j.vetimm.2015.12.004 27260249

[B39] Fernandes L. G. Siqueira G. H. Teixeira A. R. F. Silva L. P. Figueredo J. M. Cosate M. R.. (2016b). Leptospira Spp.: Novel Insights Into Host–Pathogen Interactions. Vet. Immunol. Immunopathol. 176, 50–57. doi: 10.1016/j.vetimm.2015.12.004 26727033

[B40] Fernandes L. G. V. Vieira M. L. Alves I. J. de Morais Z. M. Vasconcellos S. A. Romero E. C.. (2014). Functional and Immunological Evaluation of Two Novel Proteins of Leptospira Spp. Microbiology 160, 149–164. doi: 10.1099/mic.0.072074-0 24162609

[B41] Fernandes L. G. V. Vieira M. L. Kirchgatter K. Alves I. J. de Morais Z. M. Vasconcellos S. A.. (2012). OmpL1 Is an Extracellular Matrix- and Plasminogen-Interacting Protein of Leptospira Spp. Infect. Immun. 80, 3679–3692. doi: 10.1128/IAI.00474-12 22802342 PMC3457549

[B42] Figueira C. Croda J. Choy H. A. Haake D. A. Reis M. G. Ko A. I.. (2011). Heterologous Expression of Pathogen-Specific Genes ligA and ligB in the Saprophyte Leptospira Biflexa Confers Enhanced Adhesion to Cultured Cells and Fibronectin. BMC Microbiol. 11, 129. doi: 10.1186/1471-2180-11-129 21658265 PMC3133549

[B43] Finn M. A. Jenkin H. M. (1973). Cytopathic Effects of Leptospira Serotypes Patoc and Canicola in Three Kidney Cell Culture Systems. Am. J. Vet. Res. 34, 669–672. 4634065

[B44] Gallin W. J. (1998). Evolution of the “Classical” Cadherin Family of Cell Adhesion Molecules in Vertebrates. Mol. Biol. Evol. 15, 1099–1107. doi: 10.1093/oxfordjournals.molbev.a026017 9729874

[B45] García B. Merayo-Lloves J. Martin C. Alcalde I. Quirós L. M. Vazquez F. (2016). Surface Proteoglycans as Mediators in Bacterial Pathogens Infections. Front. Microbiol. 7, 220. doi: 10.3389/fmicb.2016.00220 26941735 PMC4764700

[B46] Ghosh K. K. Prakash A. Dhara A. Hussain M. S. Shrivastav P. Kumar P.. (2019). Role of Supramolecule ErpY-Like Lipoprotein of Leptospira in Thrombin-Catalyzed Fibrin Clot Inhibition and Binding to Complement Factors H and I, and Its Diagnostic Potential. Infect. Immun. 87, e00536–e00519. doi: 10.1128/IAI.00536-19 31548314 PMC6867842

[B47] Goeijenbier M. Gasem M. H. Meijers J. C. M. Hartskeerl R. A. Ahmed A. Goris M. G. A.. (2015). Markers of Endothelial Cell Activation and Immune Activation Are Increased in Patients With Severe Leptospirosis and Associated With Disease Severity. J. Infect. 71, 437–446. doi: 10.1016/j.jinf.2015.05.016 26048204

[B48] Gómez R. M. Vieira M. L. Schattner M. Malaver E. Watanabe M. M. Barbosa A. S.. (2008). Putative Outer Membrane Proteins of Leptospira Interrogans Stimulate Human Umbilical Vein Endothelial Cells (HUVECS) and Express During Infection. Microb. Pathog. 45, 315–322. doi: 10.1016/j.micpath.2008.08.004 18778767

[B49] Haake D. A. Levett P. N. (2015). “Leptospirosis in Humans” in Leptospira and Leptospirosis. (Berlin: Springer). doi: 10.1007/978-3-662-45059-8_5

[B50] Harrington D. D. Sleight S. D. (1966). Leptospira Pomona in Tissue Culture: Preliminary Study. Am. J. Vet. Res. 27, 249–256. 5913031

[B51] Hay E. D. (1991). Cell Biology of Extracellular Matrix (Boston, MA: Springer US). doi: 10.1007/978-1-4615-3770-0

[B52] Hoke D. E. Egan S. Cullen P. A. Adler B. (2008). LipL32 Is an Extracellular Matrix-Interacting Protein of Leptospira Spp. And Pseudoalteromonas Tunicata. Infect. Immun. 76, 2063–2069. doi: 10.1128/IAI.01643-07 18285490 PMC2346718

[B53] Hsieh C.-L. Chang E. Tseng A. Ptak C. Wu L.-C. Su C.-L.. (2016). Leptospira Immunoglobulin-Like Protein B (LigB) Binds to Both the C-Terminal 23 Amino Acids of Fibrinogen αc Domain and Factor XIII: Insight Into the Mechanism of LigB-Mediated Blockage of Fibrinogen α Chain Cross-Linking. PloS Negl. Trop. Dis. 10, e0004974. doi: 10.1371/journal.pntd.0004974 27622634 PMC5021285

[B54] Hsieh C.-L. Tseng A. He H. Kuo C.-J. Wang X. Chang Y.-F. (2017). Leptospira Immunoglobulin-Like Protein B Interacts With the 20th Exon of Human Tropoelastin Contributing to Leptospiral Adhesion to Human Lung Cells. Front. Cell. Infect. Microbiol. 7, 163. doi: 10.3389/fcimb.2017.00163 28536676 PMC5422739

[B55] Huang B. Bao L. Zhong Q. Shang Z.-L. Zhang H.-D. Wang Z.-P. (2008). Recombinant Plasmid Constructed and Cytotoxicity Studied for Outer Membrane Protein LipL32 Gene of Leptospira Strain 017. Sichuan. Da. Xue. Xue. Bao. Yi. Xue. Ban. 39, 347–350. 18575312

[B56] Isaacs R. D. (1994). *Borrelia Burgdorferi* Bind to Epithelial Cell Proteoglycans. J. Clin. Invest. 93, 809–819. doi: 10.1172/JCI117035 8113413 PMC293936

[B57] Ito T. Yanagawa R. (1987). Leptospiral Attachment to Extracellular Matrix of Mouse Fibroblast (L929) Cells. Vet. Microbiol. 15, 89–96. doi: 10.1016/0378-1135(87)90133-7 3439019

[B58] Kim D. D. Song W.-C. (2006). Membrane Complement Regulatory Proteins. Clin. Immunol. 118, 127–136. doi: 10.1016/j.clim.2005.10.014 16338172

[B59] King A. M. Bartpho T. Sermswan R. W. Bulach D. M. Eshghi A. Picardeau M.. (2013). Leptospiral Outer Membrane Protein LipL41 Is Not Essential for Acute Leptospirosis But Requires a Small Chaperone Protein, Lep, for Stable Expression. Infect. Immun. 81, 2768–2776. doi: 10.1128/IAI.00531-13 23690405 PMC3719587

[B60] Kline K. A. Fälker S. Dahlberg S. Normark S. Henriques-Normark B. (2009). Bacterial Adhesins in Host-Microbe Interactions. Cell Host Microbe 5, 580–592. doi: 10.1016/j.chom.2009.05.011 19527885

[B61] Kochi L. T. Fernandes L. G. V. Nascimento A. L. T. O. (2020). Heterologous Expression of the Pathogen-Specific LIC11711 Gene in the Saprophyte L. Biflexa Increases Bacterial Binding to Laminin and Plasminogen. Pathogens 9, 599. doi: 10.3390/pathogens9080599 PMC7460275 32707797

[B62] Kochi L. T. Fernandes L. G. V. Souza G. O. Vasconcellos S. A. Heinemann M. B. Romero E. C.. (2019). The Interaction of Two Novel Putative Proteins of Leptospira Interrogans With E-Cadherin, Plasminogen and Complement Components With Potential Role in Bacterial Infection. Virulence 10, 734–753. doi: 10.1080/21505594.2019.1650613 31422744 PMC6735628

[B63] Lähteenmäki K. Kukkonen M. Korhonen T. K. (2001). The Pla Surface Protease/Adhesin of *Yersinia Pestis* Mediates Bacterial Invasion Into Human Endothelial Cells. FEBS Lett. 504, 69–72. doi: 10.1016/S0014-5793(01)02775-2 11522299

[B64] Lambert A. Picardeau M. Haake D. A. Sermswan R. W. Srikram A. Adler B.. (2012). FlaA Proteins in Leptospira Interrogans Are Essential for Motility and Virulence But Are Not Required for Formation of the Flagellum Sheath. Infect. Immun. 80, 2019–2025. doi: 10.1128/IAI.00131-12 22451522 PMC3370569

[B65] Lee S. H. Kim S. Park S. C. Kim M. J. (2002). Cytotoxic Activities of Leptospira Interrogans Hemolysin SphH as a Pore-Forming Protein on Mammalian Cells. Infect. Immun. 70, 315–322. doi: 10.1128/IAI.70.1.315-322.2002 11748197 PMC127624

[B66] Levett P. N. (2001). Leptospirosis. Clin. Microbiol. Rev. 14, 296–326. doi: 10.1128/CMR.14.2.296-326.2001 11292640 PMC88975

[B67] Levett P. N. (2015). “Systematics of Leptospiraceae” in Leptospira and Leptospirosis. (Berlin: Springer) 11–20. doi: 10.1007/978-3-662-45059-8_2 25388130

[B68] Lima S. S. Ching A. T. C. Fávaro R. D. Da Silva J. B. Oliveira M. L. S. Carvalho E.. (2013). Adhesin Activity of Leptospira Interrogans Lipoprotein Identified by *In Vivo* and *In Vitro* Shotgun Phage Display. Biochem. Biophys. Res. Commun. 431, 342–347. doi: 10.1016/j.bbrc.2012.12.095 23291183

[B69] Lin Y.-P. Lee D.-W. McDonough S. P. Nicholson L. K. Sharma Y. Chang Y.-F. (2009). Repeated Domains of Leptospira Immunoglobulin-Like Proteins Interact With Elastin and Tropoelastin. J. Biol. Chem. 284, 19380–19391. doi: 10.1074/jbc.M109.004531 19473986 PMC2740563

[B70] Lin Y.-P. McDonough S. P. Sharma Y. Chang Y.-F. (2010). The Terminal Immunoglobulin-Like Repeats of LigA and LigB of Leptospira Enhance Their Binding to Gelatin Binding Domain of Fibronectin and Host Cells. PloS One 5, e11301. doi: 10.1371/journal.pone.0011301 20585579 PMC2892007

[B71] Lin Y.-P. McDonough S. P. Sharma Y. Chang Y.-F. (2011). Leptospira Immunoglobulin-Like Protein B (LigB) Binding to the C-Terminal Fibrinogen αc Domain Inhibits Fibrin Clot Formation, Platelet Adhesion and Aggregation. Mol. Microbiol. 79, 1063–1076. doi: 10.1111/j.1365-2958.2010.07510.x 21219469

[B72] Liu C.-Z. Shih M.-H. Tsai P.-J. (2005). ClfA221–550, a Fibrinogen-Binding Segment of Staphylococcus Aureus Clumping Factor A, Disrupts Fibrinogen Function. Thromb. Haemost. 94, 286–294. doi: 10.1160/TH05-03-0205 16113817

[B73] Longhi M. T. Oliveira T. R. Romero E. C. Gonçales A. P. de Morais Z. M. Vasconcellos S. A.. (2009). A Newly Identified Protein of Leptospira Interrogans Mediates Binding to Laminin. J. Med. Microbiol. 58, 1275–1282. doi: 10.1099/jmm.0.011916-0 19541787

[B74] Marie P. J. Haÿ E. Saidak Z. (2014). Integrin and Cadherin Signaling in Bone: Role and Potential Therapeutic Targets. Trends Endocrinol. Metab. 25, 567–575. doi: 10.1016/j.tem.2014.06.009 25034128

[B75] Marshall R. B. (1974). Ultrastructural Changes In Renal Tubules of Sheep Following Experimental Infection With Leptospira Interrogans Serotype Pomona. J. Med. Microbiol. 7, 505–508. doi: 10.1099/00222615-7-4-505 4141375

[B76] Martinez-Lopez D. G. Fahey M. Coburn J. (2010). Responses of Human Endothelial Cells to Pathogenic and Non-Pathogenic Leptospira Species. PloS Negl. Trop. Dis. 4, e918. doi: 10.1371/journal.pntd.0000918 21179504 PMC3001904

[B77] Mendes R. S. Von Atzingen M. de Morais Z. M. Gonçales A. P. Serrano S. M. T. Asega A. F.. (2011). The Novel Leptospiral Surface Adhesin Lsa20 Binds Laminin and Human Plasminogen and Is Probably Expressed During Infection. Infect. Immun. 79, 4657–4667. doi: 10.1128/IAI.05583-11 21844229 PMC3257903

[B78] Meri T. Murgia R. Stefanel P. Meri S. Cinco M. (2005). Regulation of Complement Activation at the C3-Level by Serum Resistant Leptospires. Microb. Pathog. 39, 139–147. doi: 10.1016/j.micpath.2005.07.003 16169184

[B79] Miller N. G. Froehling R. C. White R. J. (1970). Activity of Leptospires and Their Products on L Cell Monolayers. Am. J. Vet. Res. 31, 371–377. 5461150

[B80] Miller R. E. Miller N. G. White R. J. (1966). Growth of *Leptospira Pomona* and Its Effect on Various Tissue Culture Systems. J. Bacteriol. 92, 502–509. doi: 10.1128/jb.92.2.502-509.1966 16562141 PMC276269

[B81] Miller N. G. Wilson R. B. (1967). Electron Microscopic Study of the Relationship of Leptospira Pomona to the Renal Tubules of the Hamster During Acute and Chronic Leptospirosis. Am. J. Vet. Res. 92, 225–235.

[B82] Murray G. L. Srikram A. Hoke D. E. Wunder E. A. Henry R. Lo M.. (2009). Major Surface Protein LipL32 Is Not Required for Either Acute or Chronic Infection With Leptospira Interrogans. Infect. Immun. 77, 952–958. doi: 10.1128/IAI.01370-08 19103763 PMC2643616

[B83] Naiman B. M. Blumerman S. Alt D. Bolin C. A. Brown R. Zuerner R.. (2002). Evaluation of Type 1 Immune Response in Nave and Vaccinated Animals Following Challenge With Leptospira Borgpetersenii Serovar Hardjo: Involvement of WC1 ^+^ γδ and CD4 T Cells. Infect. Immun. 70, 6147–6157. doi: 10.1128/IAI.70.11.6147-6157.2002 12379692 PMC130359

[B84] Navarro P. Ruco L. Dejana E.. (1998). Differential Localization of VE- and N-Cadherins in Human Endothelial Cells: VE-Cadherin Competes with N-Cadherin for Junctional Localization. J. Cell Biol. 140, 1475–1484. doi: 10.1111/j.1365-2958.2012.07985.x 9508779 PMC2132661

[B85] Nogueira S. V. Backstedt B. T. Smith A. A. Qin J.-H. Wunder E. A. Ko A.. (2013). *Leptospira Interrogans* Enolase Is Secreted Extracellularly and Interacts With Plasminogen. PloS One 8, e78150. doi: 10.1371/journal.pone.0078150 24205133 PMC3799732

[B86] Oliveira R. de Morais Z. M. Gonçales A. P. Romero E. C. Vasconcellos S. A. Nascimento A. L. T. O. (2011). Characterization of Novel OmpA-Like Protein of *Leptospira Interrogans* That Binds Extracellular Matrix Molecules and Plasminogen. PloS One 6, e21962. doi: 10.1371/journal.pone.0021962 21755014 PMC3130794

[B87] Oliveira R. Domingos R. F. Siqueira G. H. Fernandes L. G. Souza N. M. Vieira M. L.. (2013). Adhesins of Leptospira Interrogans Mediate the Interaction to Fibrinogen and Inhibit Fibrin Clot Formation *In Vitro* . PloS Negl. Trop. Dis. 7, e2396. doi: 10.1371/journal.pntd.0002396 24009788 PMC3757074

[B88] Oliveira T. R. Longhi M. T. Gonçales A. P. de Morais Z. M. Vasconcellos S. A. Nascimento A. L. T. O. (2010). LipL53, a Temperature Regulated Protein From *Leptospira Interrogans* That Binds to Extracellular Matrix Molecules. Microbes Infect. 12, 207–217. doi: 10.1016/j.micinf.2009.12.004 20026283

[B89] Pappas C. J. Picardeau M. (2015). Control of Gene Expression in Leptospira Spp. By Transcription Activator-Like Effectors Demonstrates a Potential Role for LigA and LigB in Leptospira Interrogans Virulence. Appl. Environ. Microbiol. 81, 7888–7892. doi: 10.1128/AEM.02202-15 26341206 PMC4616954

[B90] Passalia F. J. Carvalho E. Heinemann M. B. Vieira M. L. Nascimento A. L. T. O. (2020a). The Leptospira Interrogans LIC10774 is a Multifunctional Surface Protein That Binds Calcium and Interacts With Host Components. Microbiol. Res. 235, 126470. doi: 10.1016/j.micres.2020.126470 32247916

[B91] Passalia F. J. Heinemann M. B. de Andrade S. A. Nascimento A. L. T. O. Vieira M. L. (2020b). *Leptospira Interrogans* Bat Proteins Impair Host Hemostasis by Fibrinogen Cleavage and Platelet Aggregation Inhibition. Med. Microbiol. Immunol. 209, 201–213. doi: 10.1007/s00430-020-00664-4 32078713

[B92] Passalia F. J. Heinemann M. B. Vieira M. L. Nascimento A. L. T. O. (2021). A Novel Leptospira Interrogans Protein LIC13086 Inhibits Fibrin Clot Formation and Interacts With Host Components. Front. Cell. Infect. Microbiol. 11:708739. doi: 10.3389/fcimb.2021.708739 34277477 PMC8280789

[B93] Pereira P. R. M. Fernandes L. G. V. de Souza G. O. Vasconcellos S. A. Heinemann M. B. Romero E. C.. (2017). Multifunctional and Redundant Roles of Leptospira Interrogans Proteins in Bacterial-Adhesion and Fibrin Clotting Inhibition. Int. J. Med. Microbiol. 307, 297–310. doi: 10.1016/j.ijmm.2017.05.006 28600123

[B94] Pinne M. Choy H. A. Haake D. A. (2010). The OmpL37 Surface-Exposed Protein Is Expressed by Pathogenic Leptospira During Infection and Binds Skin and Vascular Elastin. PloS Negl. Trop. Dis. 4, e815. doi: 10.1371/journal.pntd.0000815 20844573 PMC2935396

[B95] Pizarro-Cerdá J. Cossart P. (2006). Bacterial Adhesion and Entry Into Host Cells. Cell 124, 715–727. doi: 10.1016/j.cell.2006.02.012 16497583

[B96] Prozialeck W. C. Lamar P. C. Appelt D. M. (2004). Differential Expression of E-Cadherin, Ncadherin and Beta-Catenin in Proximal and Distal Segments of the Rat Nephron. BMC Physiol. 4. doi: 10.1186/1472-6793-4-10 15147582 PMC459230

[B97] Ricklin D. Hajishengallis G. Yang K. Lambris J. D. (2010). Complement: A Key System for Immune Surveillance and Homeostasis. Nat. Immunol. 11, 785–797. doi: 10.1038/ni.1923 20720586 PMC2924908

[B98] Ristow P. Bourhy P. McBride F. W. da C. Figueira C. P. Huerre M.. (2007). The OmpA-Like Protein Loa22 Is Essential for Leptospiral Virulence. PloS Pathog. 3, e97. doi: 10.1371/journal.ppat.0030097 17630832 PMC1914066

[B99] Robbins G. T. Hahn B. L. Evangelista K. V. Padmore L. Aranda P. S. Coburn J. (2015). Evaluation of Cell Binding Activities of Leptospira ECM Adhesins. PloS Negl. Trop. Dis. 9, e0003712. doi: 10.1371/journal.pntd.0003712 25875373 PMC4397020

[B100] Rose G. W. Eveland W. C. Ellinghausen H. C. (1966). Mechanisms of Tissue Cell Penetration by Leptospira Pomona: Phagocytosis of Leptospires *In Vitro* . Am. J. Vet. Res. 27, 503–511. 5959400

[B101] Rossini A. D. Teixeira A. F. Souza Filho A. Souza G. O. Vasconcellos S. A. Heinemann M. B.. (2020). Identification of a Novel Protein in the Genome Sequences of Leptospira Interrogans With the Ability to Interact With Host’s Components. J. Microbiol. Immunol. Infect. 53, 163–175. doi: 10.1016/j.jmii.2018.12.012 30713004

[B102] Rostand K. S. Esko J. D. (1997). Microbial Adherence to and Invasion Through Proteoglycans. Infect. Immun. 65, 1–8. doi: 10.1128/iai.65.1.1-8.1997 8975885 PMC174549

[B103] Salazar N. Souza M.C.L.de Biasioli A. G. Silva L.B.da Barbosa A. S. (2017). The Multifaceted Roles of Leptospira Enolase. Res. Microbiol. 168, 157–164. doi: 10.1016/j.resmic.2016.10.005 27989763

[B104] Santos J. V. Pereira P. R. M. Fernandes L. G. V. Siqueira G. H. de Souza G. O. Souza Filho A.. (2018). Binding of Human Plasminogen by the Lipoprotein LipL46 of Leptospira Interrogans. Mol. Cell. Probes 37, 12–21. doi: 10.1016/j.mcp.2017.10.004 29108931

[B105] Sato H. Coburn J. (2017). Leptospira Interrogans Causes Quantitative and Morphological Disturbances in Adherens Junctions and Other Biological Groups of Proteins in Human Endothelial Cells. PloS Negl. Trop. Dis. 11, e0005830. doi: 10.1371/journal.pntd.0005830 28750011 PMC5549773

[B106] Sebastián I. Okura N. Humbel B. M. Xu J. Hermawan I. Matsuura C.. (2021). Disassembly of the Apical Junctional Complex During the Transmigration of Leptospira Interrogans Across Polarized Renal Proximal Tubule Epithelial Cells. Cell Microbiol. 23 (9), e13343. doi: 10.1111/cmi.13343 33864347 PMC8459228

[B107] Sette A. Rappuoli R. (2010). Reverse Vaccinology: Developing Vaccines in the Era of Genomics. Immunity 33, 530–541. doi: 10.1016/j.immuni.2010.09.017 21029963 PMC3320742

[B108] Shapiro R. S. Chavez A. Collins J. J. (2018). CRISPR-Based Genomic Tools for the Manipulation of Genetically Intractable Microorganisms. Nat. Rev. Microbiol. 16, 333–339. doi: 10.1038/s41579-018-0002-7 29599458

[B109] Shi D. Sheng A. Chi L. (2021). Glycosaminoglycan-Protein Interactions and Their Roles in Human Disease. Front. Mol. Biosci. 8:639666. doi: 10.3389/fmolb.2021.639666 33768117 PMC7985165

[B110] Silva L. P. Fernandes L. G. V. Vieira M. L. de Souza G. O. Heinemann M. B. Vasconcellos S. A.. (2016). Evaluation of Two Novel Leptospiral Proteins for Their Interaction With Human Host Components. Pathog. Dis. 74:ftw040. doi: 10.1093/femspd/ftw040 27129366

[B111] Siqueira G. H. Atzingen M. V. de Souza G. O. Vasconcellos S. A. Nascimento A. L. T. O. (2016). *Leptospira Interrogans* Lsa23 Protein Recruits Plasminogen, Factor H and C4BP From Normal Human Serum and Mediates C3b and C4b Degradation. Microbiology 162, 295–308. doi: 10.1099/mic.0.000217 26614523

[B112] Siqueira G. H. de Souza G. O. Heinemann M. B. Vasconcellos S. A. Nascimento A. L. T. O. (2017). The Role of Lsa23 to Mediate the Interaction of *Leptospira Interrogans* With the Terminal Complement Components Pathway. Microb. Pathog. 112, 182–189. doi: 10.1016/j.micpath.2017.09.058 28963011

[B113] Siqueira G. H. Teixeira A. F. Fernandes L. G. de Souza G. O. Kirchgatter K. Romero E. C.. (2015). The Recombinant LIC10508 is a Plasma Fibronectin, Plasminogen, Fibrinogen and C4BP- Binding Protein of *Leptospira Interrogans* . Pathog. Dis. 74, ftv118. doi: 10.1093/femspd/ftv118 26657108

[B114] Siqueira G. H. Vasconcellos S. A. Alves I. J. de Morais Z. M. Atzingen M. V. Nascimento A. L. T. O. (2013). Characterization of Three Novel Adhesins of *Leptospira Interrogans* . Am. J. Trop. Med. Hyg. 89, 1103–1116. doi: 10.4269/ajtmh.13-0205 23958908 PMC3854887

[B115] Souza N. M. Vieira M. L. Alves I. J. de Morais Z. M. Vasconcellos S. A. Nascimento A. L. T. O. (2012). Lsa30, a Novel Adhesin of *Leptospira Interrogans* Binds Human Plasminogen and the Complement Regulator C4bp. Microb. Pathog. 53, 125–134. doi: 10.1016/j.micpath.2012.06.001 22732096

[B116] Sterling C. R. Thiermann A. B. (1981). Urban Rats as Chronic Carriers of Leptospirosis: An Ultrastructural Investigation. Vet. Pathol. 18, 628–637. doi: 10.1177/030098588101800508 7281461

[B117] Stevenson B. Choy H. A. Pinne M. Rotondi M. L. Miller M. C. DeMoll E.. (2007). *Leptospira Interrogans* Endostatin-Like Outer Membrane Proteins Bind Host Fibronectin, Laminin and Regulators of Complement. PloS One 2, e1188. doi: 10.1371/journal.pone.0001188 18000555 PMC2063517

[B118] Takahashi M. B. Teixeira A. F. Nascimento A. L. T. O. (2021). The Leptospiral LipL21 and LipL41 Proteins Exhibit Broad Spectrum of Interactions With Host Cell Components. Virulence. doi: 10.1080/21505594.2021.1993427 PMC8632080 34719356

[B119] Teixeira A. F. de Morais Z. M. Kirchgatter K. Romero E. C. Vasconcellos S. A. Nascimento A. L. T. O. (2015). Features of Two New Proteins With OmpA-Like Domains Identified in the Genome Sequences of *Leptospira Interrogans* . PloS One 10, e0122762. doi: 10.1371/journal.pone.0122762 25849456 PMC4388678

[B120] Thomas D. D. Higbie L. M. (1990). *In Vitro* Association of Leptospires With Host Cells. Infect. Immun. 58, 581–585. doi: 10.1128/iai.58.3.581-585.1990 2307512 PMC258504

[B121] Toma C. Murray G. L. Nohara T. Mizuyama M. Koizumi N. Adler B.. (2014). Leptospiral Outer Membrane Protein LMB216 is Involved in Enhancement of Phagocytic Uptake by Macrophages. Cell. Microbiol. 16, 1366–1377. doi: 10.1111/cmi.12296 24655538

[B122] Tsuchimoto M. Niikura M. Ono E. Kida H. Yanagawa R. (1984). Leptospiral Attachment to Cultured Cells. Zentralblatt Für Bakteriol. Mikrobiol. Und Hyg. Ser. A Med. Microbiol. Infect. Dis. Virol. Parasitol. 258, 268–274. doi: 10.1016/S0176-6724(84)80044-9 6532022

[B123] Verma V. Goyal M. Kala D. Gupta S. Kumar D. Kaushal A. (2020). Recent Advances in the Diagnosis of Leptospirosis. Front. Biosci. 25. doi: 10.2741/4872 32114449

[B124] Verma A. Hellwage J. Artiushin S. Zipfel P. F. Kraiczy P. Timoney J. F.. (2006). LfhA, a Novel Factor H-Binding Protein of Leptospira Interrogans. Infect. Immun. 74, 2659–2666. doi: 10.1128/IAI.74.5.2659-2666.2006 16622202 PMC1459737

[B125] Vieira M. L. Alvarez-Flores P. Kirchgatter K. Romero E. C. Alves I. J. De Morais Z. M.. (2013). Interaction of Leptospira Interrogans With Human Proteolytic Systems Enhances Dissemination Through Endothelial Cells and Protease Levels. Infect. Immun. 81, 1764–1774. doi: 10.1128/IAI.00020-13 23478319 PMC3648023

[B126] Vieira M. L. Atzingen M. V. Oliveira R. Mendes R. S. Domingos R. F. Vasconcellos S. A.. (2012). Plasminogen Binding Proteins and Plasmin Generation on the Surface of Leptospira Spp.: The Contribution to the Bacteria-Host Interactions. J. Biomed. Biotechnol. 2012, 758513. doi: 10.1155/2012/758513 23118516 PMC3481863

[B127] Vieira M. L. Atzingen M. V. Oliveira T. R. Oliveira R. Andrade D. M. Vasconcellos S. A.. (2010). *In Vitro* Identification of Novel Plasminogen-Binding Receptors of the Pathogen Leptospira Interrogans. PLoS One 5, e11259. doi: 10.1371/journal.pone.0011259 20582320 PMC2889836

[B128] Vieira M. L. D’Atri L. P. Schattner M. Habarta A. M. Barbosa A. S. de Morais Z. M.. (2007). A Novel Leptospiral Protein Increases ICAM-1 and E-Selectin Expression in Human Umbilical Vein Endothelial Cells. FEMS Microbiol. Lett. 276, 172–180. doi: 10.1111/j.1574-6968.2007.00924.x 17956423

[B129] Vieira M. L. Fernandes L. G. Domingos R. F. Oliveira R. Siqueira G. H. Souza N. M.. (2014). Leptospiral Extracellular Matrix Adhesins as Mediators of Pathogen-Host Interactions. FEMS Microbiol. Lett. 352, 129–139. doi: 10.1111/1574-6968.12349 24289724

[B130] Vieira M. L. Nascimento A. L. T. O. (2016). Interaction of Spirochetes With the Host Fibrinolytic System and Potential Roles in Pathogenesis. Crit. Rev. Microbiol. 42, 573–587. doi: 10.3109/1040841X.2014.972336 25914944

[B131] Vieira M. L. Pimenta D. C. de Morais Z. M. Vasconcellos S. A. Nascimento A. L. T. O. (2009). Proteome Analysis of Leptospira Interrogans Virulent Strain. Open Microbiol. J. 3, 69–74. doi: 10.2174/1874285800903010069 19590580 PMC2698427

[B132] Vincent A. T. Schiettekatte O. Goarant C. Neela V. K. Bernet E. Thibeaux R.. (2019). Revisiting the Taxonomy and Evolution of Pathogenicity of the Genus Leptospira Through the Prism of Genomics. PloS Negl. Trop. Dis. 13, e0007270. doi: 10.1371/journal.pntd.0007270 31120895 PMC6532842

[B133] Vinh T. Faine S. Adler B. (1984). Adhesion of Leptospires to Mouse Fibroblasts (L929) and its Enhancement by Specific Antibody. J. Med. Microbiol. 18, 73–85. doi: 10.1099/00222615-18-1-73 6379182

[B134] Wagenaar J. F. P. Goris M. G. A. Partiningrum D. L. Isbandrio B. Hartskeerl R. A. Brandjes D. P. M.. (2010). Coagulation Disorders in Patients With Severe Leptospirosis Are Associated With Severe Bleeding and Mortality. Trop. Med. Int. Heal. 15, 152–159. doi: 10.1111/j.1365-3156.2009.02434.x 20002620

[B135] Weisel J. W.. (2005). Fibrinogen and Fibrin. Adv. Protein Chem. 70, 247–299. doi: 10.1016/S0065-3233(05)70008-5 15837518

[B136] Wolberg A. S. Campbell A. S. (2008). Thrombin Generation, Fibrin Clot Formation and Hemostasis. Transfus. Apher. Sci. 38, 15–23. doi: 10.1016/j.transci.2007.12.005 18282807 PMC2408736

[B137] Wolff D. G. Castiblanco-Valencia M. M. Abe C. M. Monaris D. Morais Z. M. Souza G. O. (2013). Interaction of Leptospira Elongation Factor Tu With Plasminogen and Complement Factor H: A Metabolic Leptospiral Protein With Moonlighting Activities. PLoS One 8, e81818. doi:10.1371/journal.pone.0081818 24312361 10.1371/journal.pone.0081818 PMC3842364

[B138] Yam P. A. Miller N. G. White R. J. (1970). A Leptospiral Factor Producing a Cytopathic Effect on L Cells. J. Infect. Dis. 122, 310–317. doi: 10.1093/infdis/122.4.310 5504712

[B139] Zhang K. Murray G. L. Seemann T. Srikram A. Bartpho T. Sermswan R. W.. (2013). Leptospiral LruA Is Required for Virulence and Modulates an Interaction With Mammalian Apolipoprotein AI. Infect. Immun. 81, 3872–3879. doi: 10.1128/IAI.01195-12 23918777 PMC3811782

[B140] Zhang L. Zhang C. Ojcius D. M. Sun D. Zhao J. Lin X.. (2012). The Mammalian Cell Entry (Mce) Protein of Pathogenic Leptospira Species is Responsible for RGD Motif-Dependent Infection of Cells and Animals. Mol. Microbiol. 83, 1006–1023. doi: 10.1111/j.1365-2958.2012.07985.x 22329803

