# Supplementary material for: A Review on Host-Leptospira Interactions: What We Know and Future Expectations
Source: Front Cell Infect Microbiol. 2021 Nov 25;11:777709. doi: 10.3389/fcimb.2021.777709 (PMC8657130; doi:10.3389/fcimb.2021.777709)
Supplement: Supplementary file 1 [file Table_1.doc]

Supplementary Table 1. Features of predicted leptospiral coding sequences involved in host interactions

| **Gene ID**  **(former annotation)** | **Gene ID**  **(current annotation)** | **Ac. Number** | **Given name** | **Ligand** | **References** |
| --- | --- | --- | --- | --- | --- |
| LIC12906 | LIC_RS14935 | [WP_001049971.1](https://www.ncbi.nlm.nih.gov/protein/WP_001049971.1/) | Lsa24  LfhA  LenA | Laminin  Factor H | (Barbosa et al., 2006)  (Verma et al., 2006)  (Stevenson et al., 2007) |
| LIC10365 | LIC_RS01890 | [WP_000680185.1](https://www.ncbi.nlm.nih.gov/protein/WP_000680185.1/) | rLIC10365 | Huvec | (Vieira et al., 2007) |
| LIC10997 | LIC_RS05145 | [WP_001982778.1](https://www.ncbi.nlm.nih.gov/protein/WP_001982778.1/) | LenB | Fibronectin  Factor H  Laminin | (Stevenson et al., 2007) |
|  |  |  |  |  |  |
| LIC13006 | LIC_RS15465 | [WP_001983274.1](https://www.ncbi.nlm.nih.gov/protein/WP_001983274.1/) | LenC | Laminin  Fibronectin | Stevenson et al., 2007) |
| LIC12315 | LIC_RS11835 | [WP_000836871.1](https://www.ncbi.nlm.nih.gov/protein/WP_000836871.1/) | LenD |  |  |
| LIC13467 | LIC_RS17825 | [WP_000282726.1](https://www.ncbi.nlm.nih.gov/protein/WP_000282726.1/) | LenE |  |  |
| LIC13248 | LIC_RS16705 | [WP_000696002.1](https://www.ncbi.nlm.nih.gov/protein/WP_000696002.1/) | LenF |  |  |
| LIC10465 | LIC_RS02420 | [WP_002093809.1](https://www.ncbi.nlm.nih.gov/protein/WP_002093809.1/) | LigA | Laminin  Factor H  C4BP | (Choy et al., 2007)  (Castiblanco-Valencia et al., 2012) |
| LIC10464 | LIC_RS02415 | [WP_000717958.1](https://www.ncbi.nlm.nih.gov/protein/WP_000717958.1/) | LigB | Laminin  Fibrinogen  Factor H  C4BP | (Choy et al., 2007)  (Hsieh et al., 2016)  (Castiblanco-Valencia et al., 2012) |
| LIC10509 | LIC_RS02655 | [WP_001033571.1](https://www.ncbi.nlm.nih.gov/protein/WP_001033571.1/) | rLIC10509 | Huvec | (Gómez et al., 2008) |
| LIC10507 | LIC_RS02645 | [WP_001224416.1](https://www.ncbi.nlm.nih.gov/protein/WP_001224416.1/) | rLIC10507 | Huvec  Plasminogen | (Gómez et al., 2008)  (Siqueira et al., 2016) |
| LIC10508 | LIC_RS02650 | [WP_000721317.1](https://www.ncbi.nlm.nih.gov/protein/WP_000721317.1/) | rLIC10508 | Huvec  Fibrinogen  C4BP  Plasminogen  Plasma fibronectin |  |
| LIC10368 | LIC_RS01900 | [WP_000875567.1](https://www.ncbi.nlm.nih.gov/protein/WP_000875567.1/) | Lsa21 | Laminin  Collagen IV  Fibronectin | (Atzingen et al., 2008) |
| LIC11352 | LIC_RS06940 | [WP_000736494.1](https://www.ncbi.nlm.nih.gov/protein/WP_000736494.1/) | LipL32 | Laminin  Huvec  Plasminogen | (Hoke et al., 2008)  (Sun et al., 2010)  (Vieira et al., 2010a) |
| LIC12690 | LIC_RS13785 | [WP_002084608.1](https://www.ncbi.nlm.nih.gov/protein/WP_002084608.1/) | Lp95 | Laminin  Huvec | (Atzingen et al., 2009) |
| LIC12895 | LIC_RS14875 | [WP_000734508.1](https://www.ncbi.nlm.nih.gov/protein/WP_000734508.1/) | Lsa27 | Laminin | (Longhi et al., 2009) |
| LIC12099 | LIC_RS10715 | [WP_000252841.1](https://www.ncbi.nlm.nih.gov/protein/WP_000252841.1/) | LipL53 | Laminin  Collagen IV  Fibronectin | (Oliveira et al., 2010) |
| LIC10314 | LIC_RS01630 | [WP_000488503.1](https://www.ncbi.nlm.nih.gov/protein/WP_000488503.1/) | Lsa63 | Laminin  Collagen IV | (Vieira et al., 2010b) |
| LIC10011 | LIC_RS00055 | [WP_000610519.1](https://www.ncbi.nlm.nih.gov/protein/WP_000610519.1/) | LipL21 | Plasminogen  Elastin  Collagen I  Collagen IV  Laminin  Plasma Fibronectin  E-cadherin  Fibrinogen  Vitronectin | (Vieira et al., 2010a)  (Takahashi, 2021 submitted for publication) |
| LIC10793 | LIC_RS04090 | [WP_001087680.1](https://www.ncbi.nlm.nih.gov/protein/WP_001087680.1/) | Lp49 | Plasminogen | (Vieira et al., 2010a) |
| LIC12892 | LIC_RS14860 | [WP_000474070.1](https://www.ncbi.nlm.nih.gov/protein/WP_000474070.1/) | Lp29 |  |  |
| LIC10091 | LIC_RS00470 | [WP_000468362.1](https://www.ncbi.nlm.nih.gov/protein/WP_000468362.1/) | LipL40 |  |  |
| LIC10054 | LIC_RS00275 | [WP_033108072.1](https://www.ncbi.nlm.nih.gov/protein/WP_033108072.1/) | MPL36 |  |  |
| LIC10494 | LIC_RS02570 | [WP_001072501.1](https://www.ncbi.nlm.nih.gov/protein/WP_001072501.1/) | rLIC10494 |  |  |
| LIC12739 | LIC_RS14005 | WP_000545443.1 | rLIC12730 |  |  |
| LIC12238 | LIC_RS11435 | [WP_000859768.1](https://www.ncbi.nlm.nih.gov/protein/WP_000859768.1/) | rLIC12238 |  |  |
| LIC12263 | LIC_RS11560 | [WP_000949351.1](https://www.ncbi.nlm.nih.gov/protein/WP_000949351.1/) | OmpL37 | Fibrinogen  Laminin  Plasma fibronectin  Aortic elastin  Collagen III | (Pinne et al., 2010) |
| LIC13050 | LIC_RS15690 | [WP_000678902.1](https://www.ncbi.nlm.nih.gov/protein/WP_000678902.1/) | OmpL47 | Fibrinogen  Laminin  Plasma fibronectin  Collagen III  Aortic elastin  Skin elastin | (Pinne et al., 2010) |
| LIC10258 | LIC_RS01325 | [WP_001243368.1](https://www.ncbi.nlm.nih.gov/protein/WP_001243368.1/) | Lsa66 | Plasma fibronectin  Laminin  Plasminogen | (Oliveira et al., 2011) |
| LIC12880 | LIC_RS14785 | [WP_000034952.1](https://www.ncbi.nlm.nih.gov/protein/WP_000034952.1/) | Lp30 | Plasminogen |  |
| LIC11469 | LIC_RS07525 | [WP_000734051.1](https://www.ncbi.nlm.nih.gov/protein/WP_000734051.1/) | Lsa20 | Laminin  Plasminogen | (Mendes et al., 2011) |
| LIC11834 | LIC_RS09355 | [WP_000615236.1](https://www.ncbi.nlm.nih.gov/protein/WP_000615236.1/) | Lsa33 | Laminin  Fibrinogen  Plasminogen  C4BP | (Domingos et al., 2012) (Oliveira et al., 2013a) |
| LIC12253 | LIC_RS11510 | [WP_000760764.1](https://www.ncbi.nlm.nih.gov/protein/WP_000760764.1/) | Lsa25 | Laminin  Plasminogen  C4BP |  |
| LIC10973 | LIC_RS05025 | [WP_002072562.1](https://www.ncbi.nlm.nih.gov/protein/WP_002072562.1/) | OmpL1 | Laminin  Ea.hy926  Hep-2  Fibrinogen | (Fernandes et al., 2012) (Robbins et al., 2015) |
| LIC11087 | LIC_RS05615 | [WP_000620882.1](https://www.ncbi.nlm.nih.gov/protein/WP_000620882.1/) | Lsa30 | Laminin  Plasma Fibronectin  Plasminogen  C4BP  Fibrinogen | (Oliveira et al., 2013b)  (Souza et al., 2012) |
| LIC11360 | LIC_RS06980 | [WP_000108058.1](https://www.ncbi.nlm.nih.gov/protein/WP_000108058.1/) | Lsa23 | Laminin  Plasminogen  Fibrinogen  C8  C9  C4BP  Factor H  Fibronectin  Collagen I | (Siqueira et al., 2013, 2016, 2017) |
| LIC11009 | LIC_RS05210 | [WP_000472806.1](https://www.ncbi.nlm.nih.gov/protein/WP_000472806.1/) | Lsa26 | Laminin  Plasminogen | (Siqueira et al., 2013) |
| LIC11975 | LIC_RS10085 | [WP_001091420.1](https://www.ncbi.nlm.nih.gov/protein/WP_001091420.1/) | Lsa36 | Laminin  Plasminogen  Fibronectin |  |
| LIC12976 | LIC_RS15310 | [WP_000684307.1](https://www.ncbi.nlm.nih.gov/protein/WP_000684307.1/) | rLIC12976 | Laminin  A31  LLC-P1  Vero cells | (Lima et al., 2013) |
| LIC10645 | LIC_RS03330 | [WP_000812418.1](https://www.ncbi.nlm.nih.gov/protein/WP_000812418.1/) | Lsa44 | Laminin  Plasminogen | (Fernandes et al., 2014) |
| LIC10731 | LIC_RS03780 | [WP_000742170.1](https://www.ncbi.nlm.nih.gov/protein/WP_000742170.1/) | Lsa45 |  |  |
| LIC11574 | LIC_RS08065 | [WP_000865319.1](https://www.ncbi.nlm.nih.gov/protein/WP_000865319.1/) | rLIC11574 | E-cadherin  Ea.hy926 | (Evangelista et al., 2014b) |
| LIC13411 | LIC_RS17550 | [WP_000175921.1](https://www.ncbi.nlm.nih.gov/protein/WP_000175921.1/) | rLIC13411 | E-cadherin | (Evangelista et al., 2014a) |
| LIC11947 | LIC_RS09935 | [WP_000472812.1](https://www.ncbi.nlm.nih.gov/protein/WP_000472812.1/) | LcpA | Vitronectin  C9  Factor H  C4BP | (da Silva et al., 2015) |
| LIC11089 | LIC_RS05625 | [WP_000163348.1](https://www.ncbi.nlm.nih.gov/protein/WP_000163348.1/) | Lsa32 | Laminin  Plasminogen | (Domingos et al., 2015) |
| LIC10821 | LIC_RS04235 | [WP_000809392.1](https://www.ncbi.nlm.nih.gov/protein/WP_000809392.1/) | Lsa37 | Fibrinogen  Laminin  Plasminogen | (Silva et al., 2016) |
| LIC13059 | LIC_RS15745 | [WP_000672495.1](https://www.ncbi.nlm.nih.gov/protein/WP_000672495.1/) | Lsa25.6 | Fibrinogen  Plasminogen  Laminin | (Pereira et al., 2017) |
| LIC10879 | LIC_RS04545 | [WP_000827840.1](https://www.ncbi.nlm.nih.gov/protein/WP_000827840.1/) | Lsa16 | E-cadherin  Fibrinogen  Laminin  Plasminogen |  |
| LIC11885 | LIC_RS09605 | WP_001079033.1 | LipL46 | Plasminogen | (Santos et al., 2018) |
| LIC10831 | LIC_RS04290 | [WP_001011233.1](https://www.ncbi.nlm.nih.gov/protein/WP_001011233.1/) | rLIC10831 | Adhesion to cells via E-cadherin | (Eshghi et al., 2019) |
| LIC11711 | LIC_RS08730 | [WP_001274122.1](https://www.ncbi.nlm.nih.gov/protein/WP_001274122.1/) | rLIC11711 | Collagen IV  Laminin  E-cadherin  Plasminogen  Fibrinogen  Plasma Fibronectin  Vitronectin  C8 | (Kochi et al., 2019) |
| LIC12587 | LIC_RS13250 | [WP_000143803.1](https://www.ncbi.nlm.nih.gov/protein/WP_000143803.1/) | rLIC12587 | Laminin  Plasminogen  Plasma Fibronectin  E-cadherin  Vitronectin  C7  C8  C9 |  |
| LIC13259 | LIC_RS16765 | [WP_002145703.1](https://www.ncbi.nlm.nih.gov/protein/WP_002145703.1/) | rLIC13259 | Vitronectin  C7  C8  C9  Plasminogen  Laminin | (Cavenague et al., 2019) |
| LIC10920 | LIC_RS04740 | [WP_000820064.1](https://www.ncbi.nlm.nih.gov/protein/WP_000820064.1/) | Lsa24.9 | Plasminogen  Laminin | (Rossini et al., 2020) |
| LIC11966 | LIC_RS10040 | [WP_000716642.1](https://www.ncbi.nlm.nih.gov/protein/WP_000716642.1/) | ErpY-like | Laminin  Fibronectin  Collagen I  Chondroitin sulfate A  Chondroitin sulfate B  Hyaluronic acid  Heparan sulfate  Elastin  Fibrinogen  Factor H  Factor I | (Ghosh et al., 2019) |
| LIC10774 | LIC_RS04010 | [WP_000221043.1](https://www.ncbi.nlm.nih.gov/protein/WP_000221043.1/) | rLIC10774 | Laminin  Plasminogen  Plasma Fibronectin  Thrombin  C7  C8  C9  C4BP  Fibrinogen | (Passalia et al., 2020a) |
| LIC20040 | LIC_RS18185 | [WP_001011139.1](https://www.ncbi.nlm.nih.gov/protein/WP_001011139.1/) | batA | Fibrinogen | (Passalia et al., 2020b) |
| LIC20041 | LIC_RS18190 | [WP_001070976.1](https://www.ncbi.nlm.nih.gov/protein/WP_001070976.1/) | batB | Fibrinogen |  |
| LIC13086 | LIC_RS15890 | [WP_001218040.1](https://www.ncbi.nlm.nih.gov/protein/WP_001218040.1/) | rLIC13086 | Laminin  Plasminogen  Plasma fibronectin  Fibrinogen  C4b  C5b6  C7  C8  C9  C4BP | (Passalia et al., 2021) |
| LIC12966 | LIC_RS15260 | [WP_001228945.1](https://www.ncbi.nlm.nih.gov/protein/WP_001228945.1/) | LipL41 | Plasminogen  Elastin  Collagen I  Collagen IV  Laminin  Plasma fibronectin  Cell fibronectin  E-cadherin  Fibrinogen  Vitronectin | (Takahashi et al., 2021 submitted for publication) |

Abreviations: rLIC: recombinant *Leptospira interrogans* serovar Copenhageni; LenA: leptospiral endostatin-like protein A; LfhA: leptospiral factor H-binding protein A; Lsa: leptospiral surface adhesin; OmpL: outer membrane protein *Leptospira*; Lp: leptospiral protein; LipL: lipoprotein *Leptospira*; Lig: leptospiral immunoglobulin-like; Bat: proteins which comprise von Willebrand factor (VWF) A domains; LcpA: leptospiral complement regulator-acquiring protein A; ErpY: *B. burgdorferi* Erp protein family

REFERENCES

Atzingen, M. V., Gómez, R. M., Schattner, M., Pretre, G., Gonçales, A. P., de Morais, Z. M., et al. (2009). Lp95, a novel leptospiral protein that binds extracellular matrix components and activates e-selectin on endothelial cells. *J. Infect.* 59, 264–276. doi:10.1016/j.jinf.2009.07.010.

Atzingen, M. V, Barbosa, A. S., De Brito, T., Vasconcellos, S. A., de Morais, Z. M., Lima, D. M., et al. (2008). Lsa21, a novel leptospiral protein binding adhesive matrix molecules and present during human infection. *BMC Microbiol.* 8, 8:70. doi:10.1186/1471-2180-8-70.

Barbosa, A. S., Abreu, P. A. E., Neves, F. O., Atzingen, M. V., Watanabe, M. M., Vieira, M. L., et al. (2006). A Newly Identified Leptospiral Adhesin Mediates Attachment to Laminin. *Infect. Immun.* 74, 6356–64. doi:10.1128/IAI.00460-06.

Castiblanco-Valencia, M. M., Fraga, T. R., Silva, L. B. da, Monaris, D., Abreu, P. A. E., Strobel, S., et al. (2012). Leptospiral Immunoglobulin-like Proteins Interact With Human Complement Regulators Factor H, FHL-1, FHR-1, and C4BP. *J. Infect. Dis.* 205, 995–1004. doi:10.1093/infdis/jir875.

Cavenague, M. F., Teixeira, A. F., Filho, A. S., Souza, G. O., Vasconcellos, S. A., Heinemann, M. B., et al. (2019). Characterization of a novel protein of *Leptospira interrogans* exhibiting plasminogen, vitronectin and complement binding properties. *Int. J. Med. Microbiol.* 309, 116–129. doi:10.1016/j.ijmm.2018.12.005.

Choy, H. A., Kelley, M. M., Chen, T. L., Møller, A. K., Matsunaga, J., and Haake, D. A. (2007). Physiological Osmotic Induction of *Leptospira interrogans* Adhesion: LigA and LigB Bind Extracellular Matrix Proteins and Fibrinogen. *Infect. Immun.* 75, 2441–2450. doi:10.1128/IAI.01635-06.

da Silva, L. B., Miragaia, L. dos S., Breda, L. C. D., Abe, C. M., Schmidt, M. C. B., Moro, A. M., et al. (2015). Pathogenic *Leptospira* Species Acquire Factor H and Vitronectin via the Surface Protein LcpA. *Infect. Immun.* 83, 888–897. doi:10.1128/IAI.02844-14.

Domingos, R. F., Romero, E. C., Fernandes, L. G., Vasconcellos, S. A., de Morais, Z. M., and Nascimento, A. L. T. O. (2015). Novel *Leptospira interrogans* protein Lsa32 is expressed during infection and binds laminin and plasminogen. *Microbiology* 161, 851–864. doi:10.1099/mic.0.000041.

Domingos, R. F., Vieira, M. L., Romero, E. C., Gonçales, A., de Morais, Z. M., Vasconcellos, S. A., et al. (2012). Features of two proteins of *Leptospira interrogans* with potential role in host-pathogen interactions. *BMC Microbiol.* 12. doi:10.1186/1471-2180-12-50.

Eshghi, A., Gaultney, R. A., England, P., Brûlé, S., Miras, I., Sato, H., et al. (2019). An extracellular *Leptospira interrogans* leucine‐rich repeat protein binds human E‐ and VE‐cadherins. *Cell. Microbiol.* 21, e12949. doi:10.1111/cmi.12949.

Evangelista, K., Franco, R., Schwab, A., and Coburn, J. (2014a). *Leptospira interrogans* Binds to Cadherins. *PLoS Negl. Trop. Dis.* 8, e2672. doi:10.1371/journal.pntd.0002672.

Evangelista, K. V., Hahn, B., Wunder, E. A., Ko, A. I., Haake, D. A., and Coburn, J. (2014b). Identification of Cell-Binding Adhesins of *Leptospira interrogans*. *PLoS Negl. Trop. Dis.* 8, e3215. doi:10.1371/journal.pntd.0003215.

Fernandes, L. G. V., Vieira, M. L., Alves, I. J., de Morais, Z. M., Vasconcellos, S. A., Romero, E. C., et al. (2014). Functional and immunological evaluation of two novel proteins of *Leptospira* spp. *Microbiology* 160, 149–164. doi:10.1099/mic.0.072074-0.

Fernandes, L. G. V., Vieira, M. L., Kirchgatter, K., Alves, I. J., de Morais, Z. M., Vasconcellos, S. A., et al. (2012). OmpL1 Is an Extracellular Matrix- and Plasminogen-Interacting Protein of *Leptospira* spp. *Infect. Immun.* 80, 3679–3692. doi:10.1128/IAI.00474-12.

Ghosh, K. K., Prakash, A., Dhara, A., Hussain, M. S., Shrivastav, P., Kumar, P., et al. (2019). Role of Supramolecule ErpY-Like Lipoprotein of *Leptospira* in Thrombin-Catalyzed Fibrin Clot Inhibition and Binding to Complement Factors H and I, and Its Diagnostic Potential. *Infect. Immun.* 87, e00536-19. doi:10.1128/IAI.00536-19.

Gómez, R. M., Vieira, M. L., Schattner, M., Malaver, E., Watanabe, M. M., Barbosa, A. S., et al. (2008). Putative outer membrane proteins of Leptospira interrogans stimulate human umbilical vein endothelial cells (HUVECS) and express during infection. *Microb. Pathog.* 45, 315–322. doi:10.1016/j.micpath.2008.08.004.

Hoke, D. E., Egan, S., Cullen, P. A., and Adler, B. (2008). LipL32 Is an Extracellular Matrix-Interacting Protein of *Leptospira* spp. and *Pseudoalteromonas tunicata*. *Infect. Immun.* 76, 2063–2069. doi:10.1128/IAI.01643-07.

Hsieh, C.-L., Chang, E., Tseng, A., Ptak, C., Wu, L.-C., Su, C.-L., et al. (2016). Leptospira Immunoglobulin-Like Protein B (LigB) Binds to Both the C-Terminal 23 Amino Acids of Fibrinogen αC Domain and Factor XIII: Insight into the Mechanism of LigB-Mediated Blockage of Fibrinogen α Chain Cross-Linking. *PLoS Negl. Trop. Dis.* 10, e0004974. doi:10.1371/journal.pntd.0004974.

Kochi, L. T., Fernandes, L. G. V., Souza, G. O., Vasconcellos, S. A., Heinemann, M. B., Romero, E. C., et al. (2019). The interaction of two novel putative proteins of *Leptospira interrogans* with E-cadherin, plasminogen and complement components with potential role in bacterial infection. *Virulence* 10. doi:10.1080/21505594.2019.1650613.

Lima, S. S., Ching, A. T. C., Fávaro, R. D., Da Silva, J. B., Oliveira, M. L. S., Carvalho, E., et al. (2013). Adhesin activity of *Leptospira interrogans* lipoprotein identified by in vivo and in vitro shotgun phage display. *Biochem. Biophys. Res. Commun.* 431, 342–347. doi:10.1016/j.bbrc.2012.12.095.

Longhi, M. T., Oliveira, T. R., Romero, E. C., Gonçales, A. P., de Morais, Z. M., Vasconcellos, S. A., et al. (2009). A newly identified protein of *Leptospira interrogans* mediates binding to laminin. *J. Med. Microbiol.* 58, 1275–1282. doi:10.1099/jmm.0.011916-0.

Mendes, R. S., Von Atzingen, M., de Morais, Z. M., Gonçales, A. P., Serrano, S. M. T., Asega, A. F., et al. (2011). The Novel Leptospiral Surface Adhesin Lsa20 Binds Laminin and Human Plasminogen and Is Probably Expressed during Infection. *Infect. Immun.* 79, 4657–4667. doi:10.1128/IAI.05583-11.

Oliveira, R., de Morais, Z. M., Gonçales, A. P., Romero, E. C., Vasconcellos, S. A., and Nascimento, A. L. T. O. (2011). Characterization of Novel OmpA-Like Protein of *Leptospira interrogans* That Binds Extracellular Matrix Molecules and Plasminogen. *PLoS One* 6, e21962. doi:10.1371/journal.pone.0021962.

Oliveira, R., Domingos, R. F., Siqueira, G. H., Fernandes, L. G., Souza, N. M., Vieira, M. L., et al. (2013a). Adhesins of *Leptospira interrogans* Mediate the Interaction to Fibrinogen and Inhibit Fibrin Clot Formation In Vitro. *PLoS Negl. Trop. Dis.* 7, e2396. doi:10.1371/journal.pntd.0002396.

Oliveira, R., Domingos, R. F., Siqueira, G. H., Fernandes, L. G., Souza, N. M., Vieira, M. L., et al. (2013b). Adhesins of *Leptospira interrogans* Mediate the Interaction to Fibrinogen and Inhibit Fibrin Clot Formation In Vitro. *PLoS Negl. Trop. Dis.* 7, doi:10.1371/journal.pntd.0002396. doi:10.1371/journal.pntd.0002396.

Oliveira, T. R., Longhi, M. T., Gonçales, A. P., de Morais, Z. M., Vasconcellos, S. A., and Nascimento, A. L. T. O. (2010). LipL53, a temperature regulated protein from *Leptospira interrogans* that binds to extracellular matrix molecules. *Microbes Infect.* 12, 207–217. doi:10.1016/j.micinf.2009.12.004.

Passalia, F. J., Carvalho, E., Heinemann, M. B., Vieira, M. L., and Nascimento, A. L. T. O. (2020a). The Leptospira interrogans LIC10774 is a multifunctional surface protein that binds calcium and interacts with host components. *Microbiol. Res.* 235. doi:10.1016/j.micres.2020.126470.

Passalia, F. J., Heinemann, M. B., de Andrade, S. A., Nascimento, A. L. T. O., and Vieira, M. L. (2020b). *Leptospira interrogans* Bat proteins impair host hemostasis by fibrinogen cleavage and platelet aggregation inhibition. *Med. Microbiol. Immunol.* 209, 201–213. doi:10.1007/s00430-020-00664-4.

Passalia, F. J., Heinemann, M. B., Vieira, M. L., and Nascimento, A. L. T. O. (2021). A Novel Leptospira interrogans Protein LIC13086 Inhibits Fibrin Clot Formation and Interacts With Host Components. *Front. Cell. Infect. Microbiol.* 11. doi:10.3389/fcimb.2021.708739.

Pereira, P. R. M., Fernandes, L. G. V., de Souza, G. O., Vasconcellos, S. A., Heinemann, M. B., Romero, E. C., et al. (2017). Multifunctional and Redundant Roles of *Leptospira interrogans* Proteins in Bacterial-Adhesion and fibrin clotting inhibition. *Int. J. Med. Microbiol.* 307, 297–310. doi:10.1016/j.ijmm.2017.05.006.

Pinne, M., Choy, H. A., and Haake, D. A. (2010). The OmpL37 Surface-Exposed Protein Is Expressed by Pathogenic Leptospira during Infection and Binds Skin and Vascular Elastin. *PLoS Negl. Trop. Dis.* 4, e815. doi:10.1371/journal.pntd.0000815.

Robbins, G. T., Hahn, B. L., Evangelista, K. V., Padmore, L., Aranda, P. S., and Coburn, J. (2015). Evaluation of Cell Binding Activities of Leptospira ECM Adhesins. *PLoS Negl. Trop. Dis.* 9, e0003712. doi:10.1371/journal.pntd.0003712.

Rossini, A. D., Teixeira, A. F., Souza Filho, A., Souza, G. O., Vasconcellos, S. A., Heinemann, M. B., et al. (2020). Identification of a novel protein in the genome sequences of *Leptospira interrogans* with the ability to interact with host’s components. *J. Microbiol. Immunol. Infect.* 53, 163–175. doi:10.1016/j.jmii.2018.12.012.

Santos, J. V., Pereira, P. R. M., Fernandes, L. G. V., Siqueira, G. H., de Souza, G. O., Souza Filho, A., et al. (2018). Binding of human plasminogen by the lipoprotein LipL46 of *Leptospira interrogans*. *Mol. Cell. Probes* 37, 12–21. doi:10.1016/j.mcp.2017.10.004.

Silva, L. P., Fernandes, L. G. V., Vieira, M. L., de Souza, G. O., Heinemann, M. B., Vasconcellos, S. A., et al. (2016). Evaluation of two novel leptospiral proteins for their interaction with human host components. *Pathog. Dis.* 74, ftw040. doi:10.1093/femspd/ftw040.

Siqueira, G. H., Atzingen, M. V., de Souza, G. O., Vasconcellos, S. A., and Nascimento, A. L. T. O. (2016). *Leptospira interrogans* Lsa23 protein recruits plasminogen, factor H and C4BP from normal human serum and mediates C3b and C4b degradation. *Microbiology* 162, 295–308. doi:10.1099/mic.0.000217.

Siqueira, G. H., de Souza, G. O., Heinemann, M. B., Vasconcellos, S. A., and Nascimento, A. L. T. O. (2017). The role of Lsa23 to mediate the interaction of *Leptospira interrogans* with the terminal complement components pathway. *Microb. Pathog.* 112, 182–189. doi:10.1016/j.micpath.2017.09.058.

Siqueira, G. H., Vasconcellos, S. A., Alves, I. J., de Morais, Z. M., Atzingen, M. V., and Nascimento, A. L. T. O. (2013). Characterization of Three Novel Adhesins of *Leptospira interrogans*. *Am. J. Trop. Med. Hyg.* 89, 1103–16. doi:10.4269/ajtmh.13-0205.

Souza, N. M., Vieira, M. L., Alves, I. J., de Morais, Z. M., Vasconcellos, S. A., and Nascimento, A. L. T. O. (2012). Lsa30, a novel adhesin of *Leptospira interrogans* binds human plasminogen and the complement regulator C4bp. *Microb. Pathog.* 53, 125–34. doi:10.1016/j.micpath.2012.06.001.

Stevenson, B., Choy, H. A., Pinne, M., Rotondi, M. L., Miller, M. C., DeMoll, E., et al. (2007). *Leptospira interrogans* Endostatin-Like Outer Membrane Proteins Bind Host Fibronectin, Laminin and Regulators of Complement. *PLoS One* 2, e1188. doi:10.1371/journal.pone.0001188.

Sun, Z., Bao, L., Li, D., Huang, B., and Wu, B. (2010). Effect of *Leptospira interrogans* outer membrane proteins LipL32 on HUVEC. *Microb. Pathog.* 49, 116–21. doi:10.1016/j.micpath.2010.05.006.

Verma, A., Hellwage, J., Artiushin, S., Zipfel, P. F., Kraiczy, P., Timoney, J. F., et al. (2006). LfhA, a Novel Factor H-Binding Protein of *Leptospira interrogans*. *Infect. Immun.* 74, 2659–66. doi:10.1128/IAI.74.5.2659-2666.2006.

Vieira, M. L., Atzingen, M. V., Oliveira, T. R., Oliveira, R., Andrade, D. M., Vasconcellos, S. A., et al. (2010a). In Vitro Identification of Novel Plasminogen-Binding Receptors of the Pathogen *Leptospira interrogans*. *PLoS One* 5, e11259. doi:10.1371/journal.pone.0011259.

Vieira, M. L., D’Atri, L. P., Schattner, M., Habarta, A. M., Barbosa, A. S., de Morais, Z. M., et al. (2007). A novel leptospiral protein increases ICAM-1 and E-selectin expression in human umbilical vein endothelial cells. *FEMS Microbiol. Lett.* 276, 172–80. doi:10.1111/j.1574-6968.2007.00924.x.

Vieira, M. L., de Morais, Z. M., Gonçales, A. P., Romero, E. C., Vasconcellos, S. A., and Nascimento, A. L. T. O. (2010b). Lsa63, a newly identified surface protein of *Leptospira interrogans* binds laminin and collagen IV. *J. Infect.* 60, 52–64. doi:10.1016/j.jinf.2009.10.047.
